# Untangling the Role of MYC in Sarcomas and Its Potential as a Promising Therapeutic Target

**DOI:** 10.3390/ijms26051973

**Published:** 2025-02-25

**Authors:** Fabio Sias, Stefano Zoroddu, Rossana Migheli, Luigi Bagella

**Affiliations:** 1 Department of Biomedical Sciences, University of Sassari, Viale San Pietro 43/b, 07100 Sassari, Italy; f.sias@phd.uniss.it (F.S.); szoroddu@uniss.it (S.Z.); 2 Department of Medical, Surgical and Experimental Sciences, University of Sassari, 07100 Sassari, Italy; rmigheli@uniss.it; 3 Sbarro Institute for Cancer Research and Molecular Medicine, Centre for Biotechnology, College of Science and Technology, Temple University, Philadelphia, PA 19122, USA

**Keywords:** MYC, sarcoma, rhabdomyosarcoma, osteosarcoma, synovial sarcoma, malignant rhabdoid tumor, leiomyosarcoma, Ewing’s sarcoma

## Abstract

MYC plays a pivotal role in the biology of various sarcoma subtypes, acting as a key regulator of tumor growth, proliferation, and metabolic reprogramming. This oncogene is frequently dysregulated across different sarcomas, where its expression is closely intertwined with the molecular features unique to each subtype. MYC interacts with critical pathways such as cell cycle regulation, apoptosis, and angiogenesis, amplifying tumor aggressiveness and resistance to standard therapies. Furthermore, MYC influences the tumor microenvironment by modulating cell–extracellular matrix interactions and immune evasion mechanisms, further complicating therapeutic management. Despite its well-established centrality in sarcoma pathogenesis, targeting MYC directly remains challenging due to its “undruggable” protein structure. However, emerging therapeutic strategies, including indirect MYC inhibition via epigenetic modulators, transcriptional machinery disruptors, and metabolic pathway inhibitors, offer new hope for sarcoma treatment. This review underscores the importance of understanding the intricate roles of MYC across sarcoma subtypes to guide the development of effective targeted therapies. Given MYC’s central role in tumorigenesis and progression, innovative approaches aiming at MYC inhibition could transform the therapeutic landscape for sarcoma patients, providing a much-needed avenue to overcome therapeutic resistance and improve clinical outcomes.

## 1. Introduction

Sarcomas are malignant tumors that arise from mesenchymal tissues throughout the body [1]. Although they originate from ectodermal tissue, malignant tumors of the peripheral nerves are included in this classification due to their similarities in clinical behavior, management, and outcomes [2]. Sarcomas are categorized into bone sarcomas (BSs) and soft tissue sarcomas (STSs), which can affect fibrous, cartilaginous, muscular, synovial, or adipose tissues. Despite the diversity of these tumor types, sarcomas account for less than 1% of all incident cancers in the United States, with an estimated 15,000 diagnoses in 2022 [3]. The rarity of sarcomas, combined with their extensive histological diversity and wide range of clinical behaviors [4], presents significant challenges in diagnosis and treatment. Additionally, there is an incomplete understanding of the molecular processes driving their development and progression. Advancements in this field have the potential to enhance diagnostic accuracy, identify reliable prognostic markers, and develop more effective, targeted therapies, offering hope for improved outcomes in managing these difficult-to-treat malignancies [5].

One critical area of research focuses on unraveling the genetic and epigenetic factors that contribute to these tumors, including the *MYC* gene.

**Figure 1:**
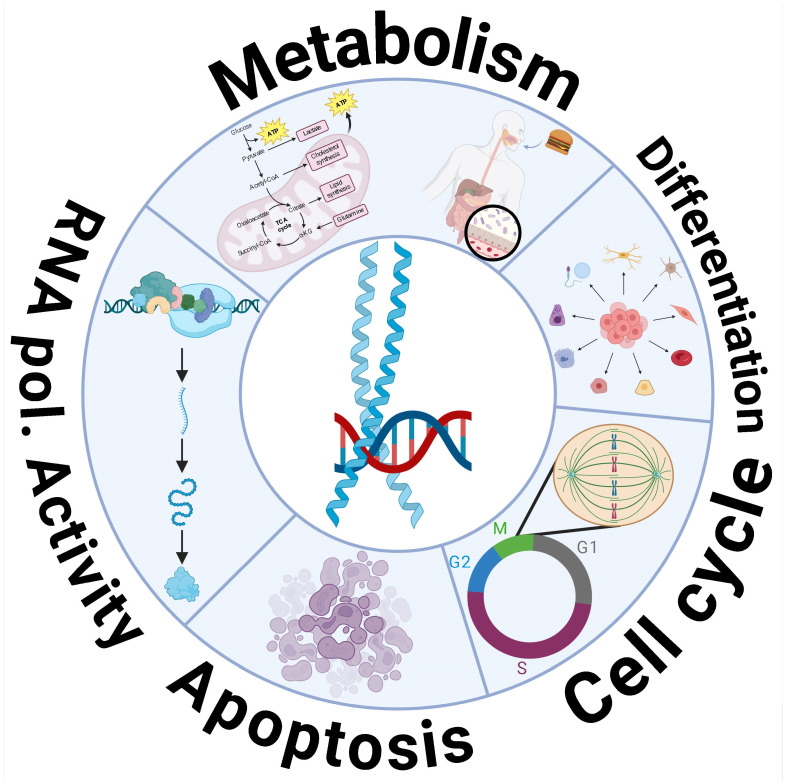
Pathways regulated by MYC activity.

The *MYC* family of proto-oncogenes is, notably, the most frequently deregulated group of genes across all human cancers. *MYC* genes are evolutionarily conserved, and in mammalian cells, three genes encode three MYC proteins: *MYC*, which is expressed in almost all tissues; *MYCN*, which is specific to neuronal contexts; and *MYCL*, which is associated with lung tissues [6]. All three proteins can perform overlapping functions [7]. MYC proteins play a key role in promoting cellular proliferation and regulating the cell cycle, along with other essential cellular activities (Figure 1). Given their critical role in overseeing cellular processes, MYC proteins are indispensable for embryonic development and the maintenance of self-renewing tissues in adults [8].

**Figure 2:**
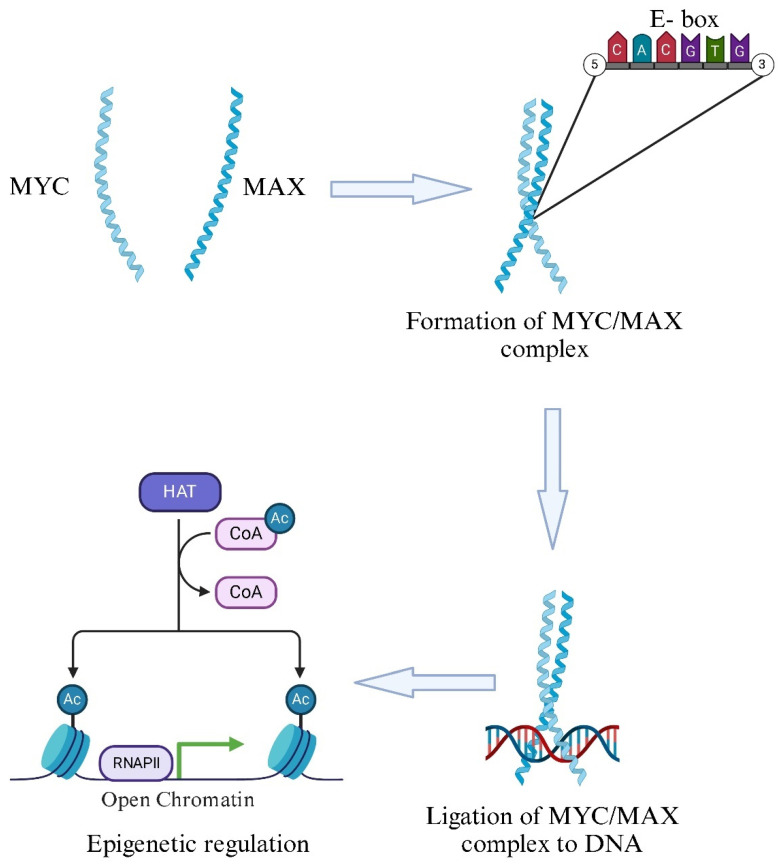
MYC/MAX leading the way to epigenetic regulation.

MYC regulates gene expression by interacting with a protein called MAX. The MYC-MAX heterodimer is the only form that can bind to DNA and activate transcription [9]; in fact, MYC does not form homodimers and remains mostly unstructured until it pairs with MAX (Figure 2) [10].

This complex targets specific DNA sequences, known as Enhancer boxes (E-boxes) (CACGTG), located in the promoters of MYC target genes. It contains a basic region essential for E-box binding and a basic helix–loop–helix leucine zipper domain (bHLHLZ), essential for dimerization with MAX [11]. The interaction between MYC and MAX induces a helical shape in the basic domain, allowing them to anchor into the major grooves of DNA. The MYC/MAX heterodimers then recruit additional cofactors to stimulate RNA polymerases I, II, and III, thereby activating the transcription of at least 15% of all genes, including those encoding both proteins and non-coding RNAs [10,12].

**Figure 3:**
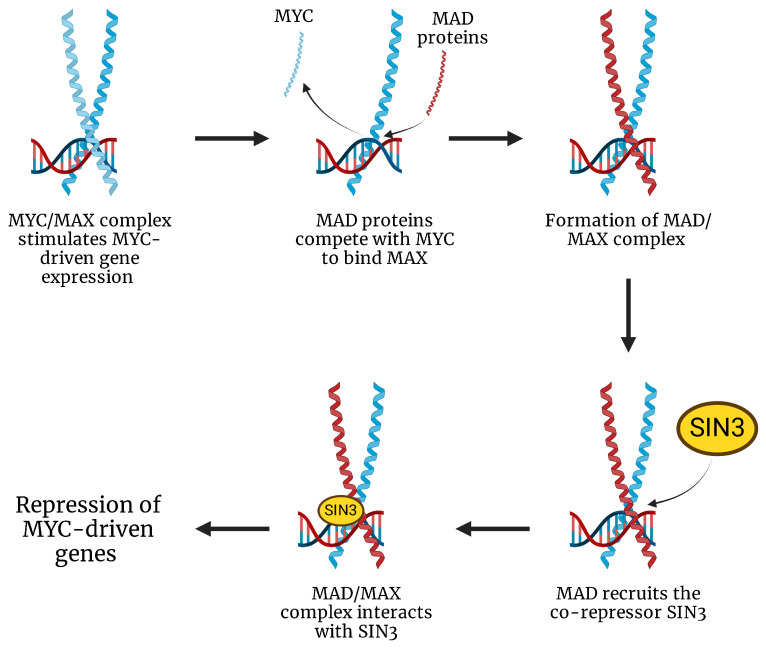
MYC activity is regulated by its natural antagonists: MAD proteins. These proteins compete with MYC for binding to MAX, forming MAD-MAX heterodimers that repress MYC-driven gene expression by recruiting co-repressors such as SIN3.

In addition to its direct interactions with MAX, MYC activity is regulated by its natural antagonists, the MAD family of proteins [13]. These proteins compete with MYC for binding to MAX, forming MAD-MAX heterodimers that repress MYC-driven gene expression by recruiting co-repressors, such as SIN3 [14]. This interaction helps to maintain a critical balance in cellular processes by limiting MYC’s proliferative activity. Disruption of this regulatory balance, such as the loss or dysfunction of MAD proteins, can result in uncontrolled MYC activity, a common feature in many cancers, including sarcomas [15]. The MAD proteins play an essential role in controlling MYC’s activity, preventing it from driving excessive cell proliferation and tumorigenesis. When this balance is altered, MYC becomes a potent oncogene that promotes cancer progression by upregulating genes involved in cell cycle regulation, metabolism, and survival (Figure 3).

This review aims to explore which sarcoma subtypes (BSs and STSs) are most associated with MYC overexpression and to investigate potential future therapeutic strategies highlighting MYC’s potential as a key therapeutic target and its influence on the development of future treatment strategies.

## 2. MYC in Sarcomas

**Figure 4:**
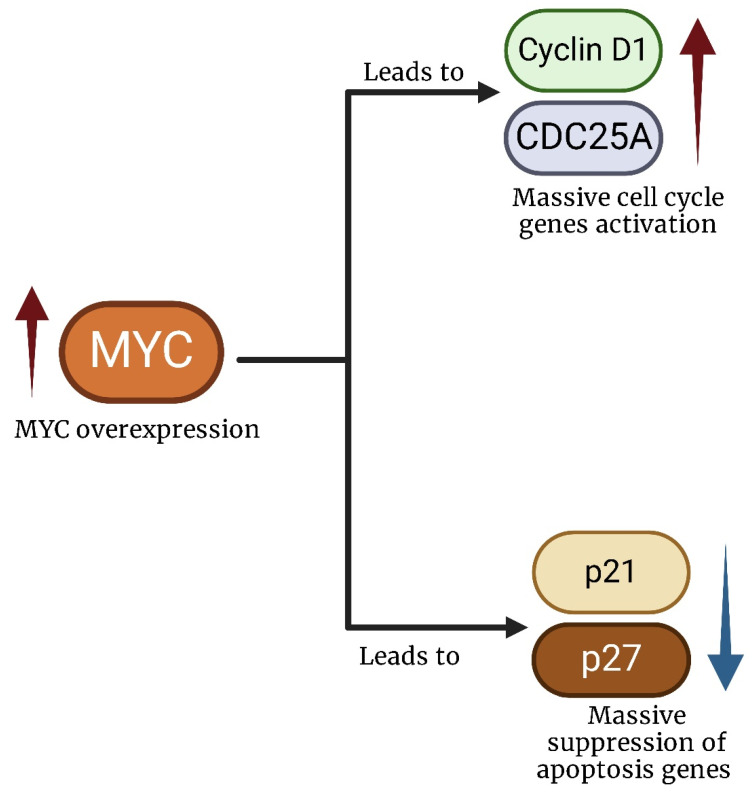
MYC’s role in cancer.

*MYC* plays a role in cancer that goes far beyond driving cell division. It has been found to influence nearly all key aspects of cancer: it promotes angiogenesis, facilitates communication with the tumor microenvironment (TME), suppresses the immune system’s ability to fight tumors, and even contributes to resistance against many standard therapies (Figure 4) [16,17,18].

**Figure 5:**
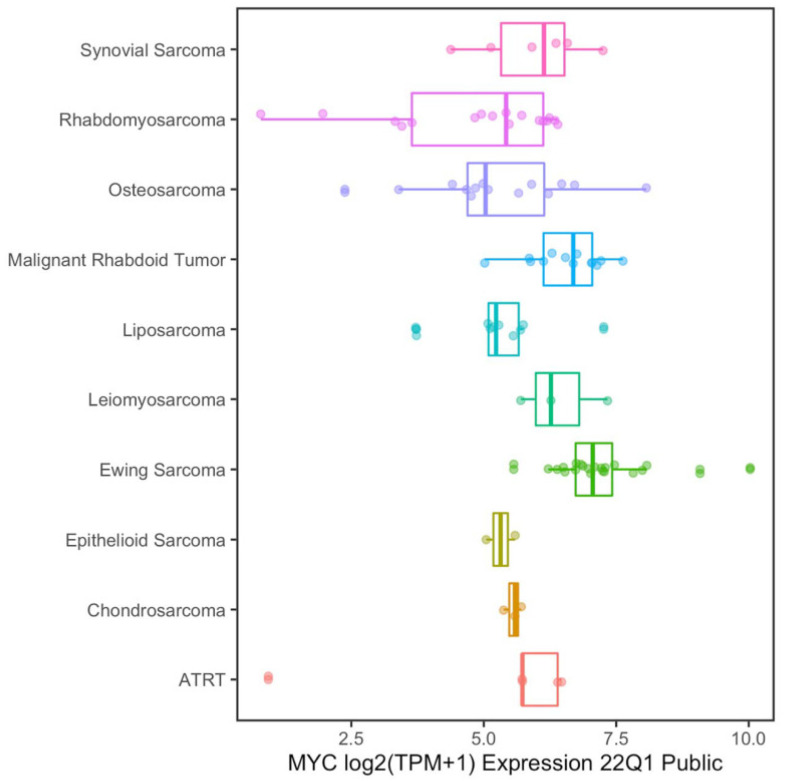
*MYC* expression in Cancer Cell Line Encyclopedia cell lines. Boxplot displays RNA-seq expression levels from CCLE cancer samples. TPM: transcripts per Kilobase Million. Extracted, and graciously shared by [17].

Unlike other genes, *MYC* is rarely mutated in tumors; it is instead frequently amplified, particularly in the chromosomal region 8q24 where it is located [16], and it is frequently associated with a poor prognosis [17]. However, previous studies have shown that in certain subtypes of sarcomas (both BSs and STSs), such as Rhabdomyosarcoma, osteosarcoma, synovial sarcoma, Malignant Rhabdoid Tumor, Leiomyosarcoma, Ewing’s sarcoma, liposarcomas, and Atypical Teratoid/Rhabdoid tumor, *MYC* is frequently amplified (Figure 5).

The amplification of *MYC* leads to an increased number of gene copies and elevated levels of the MYC protein, resulting in excessive gene activity that affects critical cellular pathways such as proliferation, angiogenesis, and metabolism. Due to these factors, MYC is considered a highly attractive target for cancer treatment and is prioritized in the search for effective cancer therapies [19].

## 3. MYC in Rhabdomyosarcoma

**Figure 6:**
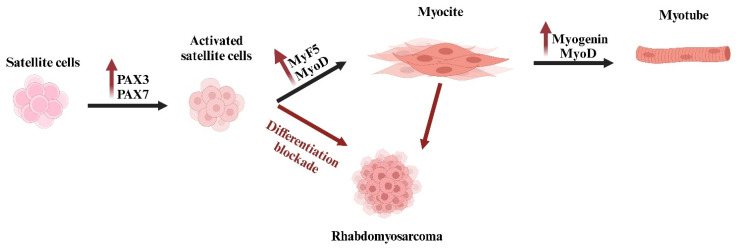
In skeletal muscle myogenesis within RMS, satellite cells expressing early myogenic markers like *MyoD* and *MyF5* are activated, continuing with the expression of *Myogenin* and *MyoD* that leads to the formation of Myotubes, much like in the normal physiological process. However, their expression levels and/or the availability of binding cofactors are often disrupted. As a result, the cell cycle fails to arrest, leading to continued proliferation at the expense of differentiation, ultimately contributing to tumor mass formation.

Rhabdomyosarcoma (RMS) is a very rare type of cancer overall, but it is one of the most common subtypes of sarcoma, particularly in children and adolescents [20]. RMS belongs to the group of sarcomas, which are cancers that develop in connective tissues. In this case, the cancer cells are believed to originate from skeletal muscle progenitor cells (Figure 6). While RMS tumors can develop at any age, they are most widely commonly found in children under 15 years old [21].

Based on their clinical and pathological features, as well as genetic variations, these tumors are classified into four subtypes: Embryonal Rhabdomyosarcoma (ERMS), Alveolar Rhabdomyosarcoma (ARMS), Pleomorphic Rhabdomyosarcoma (PRMS), and Spindle Cell/Sclerosing Rhabdomyosarcoma (SpRMS) (Table 1) [22]. The prognoses and outcomes of RMS tumors are closely linked to their subtypes, indicating that each subtype has distinct biological characteristics [23].

About 70% of RMS patients achieve a five-year survival rate with standard treatments, which typically include a combination of chemotherapy, surgery, and radiotherapy. However, the remaining 30% do not survive, often due to metastasis or tumor relapse that becomes resistant to further treatment [24].

Given the limitations of conventional therapies, researchers are exploring new treatment strategies that target specific molecular mechanisms involved in RMS. Studies have revealed that *MYC* and *MYCN* oncogenes are often amplified or overexpressed in RMS cells, making them attractive targets for these innovative therapeutic approaches [17].

Evidence strongly supports that *MYC* plays a pivotal role as an oncogenic signaling molecule in RMS [25]. Elevated levels of *MYC* are frequently linked to increased tumor aggressiveness and worse clinical outcomes [26], particularly in two of the four subtypes: ERMS, which shows lower levels of MYC, and ARMS, the subtype characterized by the highest *MYC* levels [27].

In ERMS, *MYC* acts as a pro-proliferative and anti-apoptotic factor [28], partly by repressing the cell cycle inhibitor *p21* [29]. Notably, the depletion of MYC in ERMS cell lines resulted in a reduction in markers associated with metastasis, invasion, and angiogenesis; moreover, *MYC* enhances radio-resistance in ERMS cells by protecting them from apoptosis, minimizing DNA damage, and promoting DNA repair mechanisms [30].

*MYC* plays, instead, a crucial role in the development and progression of ARMS, a highly aggressive pediatric cancer characterized by its association with specific fusion proteins, primarily PAX3-FOXO1 or PAX7-FOXO1 [31]. These fusion proteins arise from chromosomal translocations and act as oncogenic transcription factors, driving the malignant phenotype of ARMS by altering the expression of numerous target genes [31]. The interaction between *MYC* and these fusion proteins is a critical aspect of the molecular pathology of this disease [32].

In ARMS, *MYC* is frequently upregulated and contributes to the tumor’s aggressive behavior by promoting cellular proliferation, inhibiting apoptosis, and enhancing metastatic potential [33]. The fusion proteins PAX3-FOXO1 and PAX7-FOXO1 interact directly or indirectly with *MYC*-related pathways, amplifying its oncogenic effects [34]. One mechanism involves the transcriptional activation of *MYC* by these fusion proteins, which bind to enhancer regions or interact with other transcriptional coregulators to upregulate MYC expression. This upregulation enhances the tumor’s proliferative capacity and supports the metabolic reprogramming necessary for rapid growth [35].

Furthermore, MYC collaborates with the fusion proteins to suppress cell cycle inhibitors such as p21 and p27, tipping the balance in favor of unchecked proliferation [36,37]. *MYC* also modulates the tumor microenvironment, in part through its influence on angiogenic factors, allowing the tumor to sustain its growth and invasion [38]. These fusion proteins, by interacting with MYC or its downstream effectors, reinforce pathways that prevent apoptosis, including those involving BCL-2 family proteins, thereby promoting survival even under stress conditions like hypoxia or treatment-induced DNA damage [39].

A significant aspect of the MYC fusion–protein interplay in ARMS is their combined impact on the DNA damage response [40]. MYC enhances DNA repair processes, while the fusion proteins contribute to genomic instability by deregulating cell cycle checkpoints. This dual effect increases the tumor’s adaptability and resistance to therapies such as radiation and chemotherapy [41].

Targeting MYC in ARMS is challenging due to its “undruggable” nature, but recent advances have identified potential strategies to disrupt its activity or downstream effects. These include inhibitors of bromodomain-containing proteins (BET inhibitors) that indirectly suppress MYC transcription, small molecules targeting MYC-MAX dimerization, and therapies aimed at blocking the metabolic dependencies driven by *MYC*.

**Figure 7:**
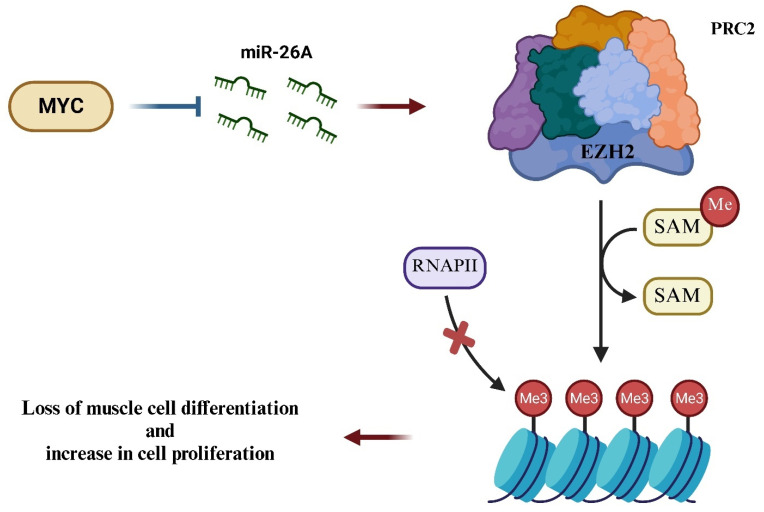
MYC promotes the expression of EZH2 by repressing miR-26a, a critical microRNA known to suppress EZH2 by directly targeting its mRNA. This dynamic illustrates how MYC fine-tunes EZH2 levels, shedding light on its broader role in regulating gene expression via the modulation of specific miRNAs.

Several studies have highlighted that MYC and Polycomb group (PcG) proteins can interact with each other, giving rise to a malignant and aggressive phenotype [42]. PcG proteins, particularly the Polycomb Repressive Complex 2 (PRC2), play a role in silencing stemness markers that regulate muscle differentiation. The catalytic subunit of PRC2, Enhancer of Zeste (EZH2), can trimethylate lysine 27 on histone H3, leading to gene silencing. EZH2 is often deregulated in ERMS and various other tumors, and in some cases, it is linked to increased tumor malignancy [19]. As an example, research published in *Blood* demonstrated that MYC stimulates the expression of EZH2 by repressing its negative regulator, miR-26a (Figure 7). This mechanism suggests that MYC can influence the expression of EZH2 by modulating specific miRNAs [42]. Additionally, a study published in *Oncotarget* observed that the upregulation of *MYC* in mouse prostate tissue led to an increase in EZH2 expression, accompanied by a decrease in miR-26a and miR-29a [43]. These findings indicate that MYC may regulate EZH2 expression through both transcriptional and post-transcriptional mechanisms.

The treatment with EZH2 inhibitor has shown potential in promoting myogenic differentiation in embryonal RMS cells. This approach could provide a promising therapeutic strategy to restore normal differentiation pathways and reduce the malignancy of RMS [44].

**Figure 8:**
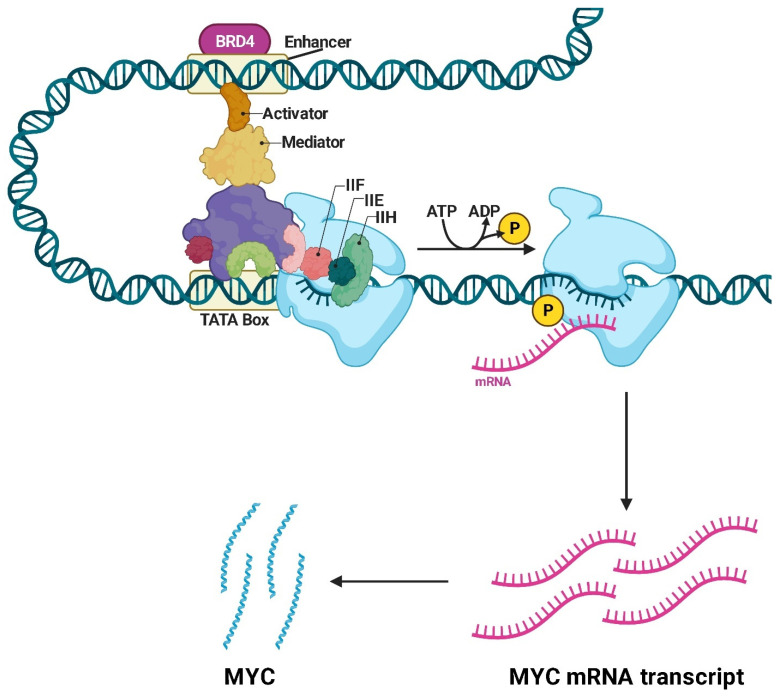
BRD4 binds to super-enhancer regions near the MYC gene, driving its transcriptional activation. This process involves the interaction of BRD4 bromodomains with acetylated lysine residues on histones, such as H3K27ac, which are enriched in MYC regulatory regions. These interactions help maintain an open chromatin structure, facilitating the recruitment of transcriptional machinery to sustain MYC expression.

Another pharmacological approach for RMS is represented by bromodomain and Extra-Terminal domain (BET) inhibitors [45]. The BET family of proteins includes members such as BRD2, BRD3, BRD4, and BRDT [46]. These proteins are characterized by the presence of bromodomains, which are structural modules capable of specifically recognizing and binding acetylated lysine residues found on histones and other proteins [47]. Among the BET proteins, BRD4 plays a crucial role in the transcriptional regulation of the oncogene *MYC* [48]. The interaction between BET proteins and MYC has both upstream and downstream effects; upstream, BRD4 binds to super-enhancer regions located near the *MYC* gene, facilitating its transcriptional activation (Figure 8). This process is mediated by the interaction of BRD4 bromodomains with acetylated lysine residues on histones, such as H3K27ac, in *MYC* regulatory regions [49]. Downstream, once MYC is expressed, it drives the transcription of numerous target genes involved in cell growth and proliferation, supporting the tumorigenic phenotype [50]. Furthermore, BRD4 amplifies MYC’s transcriptional activity through global epigenetic regulation [51].

**Figure 9:**
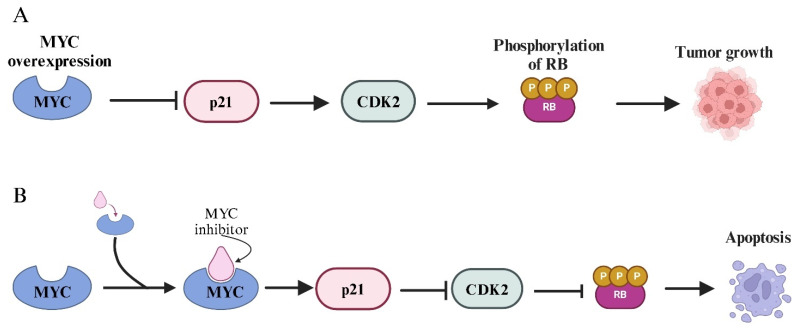
(**A**) MYC promotes RMS development by repressing p21 transcription; (**B**) treating RMS cells with a c-MYC inhibitor showed significantly increased time-dependent levels of apoptosis due to p21 activation and inhibition of CDK2.

Given the key roles of BET proteins in MYC regulation, the inhibition of BET proteins has emerged as a promising therapeutic strategy in oncology [52]. Compounds known as BET inhibitors (BETi), such as JQ1, bind to the bromodomains of BET proteins, preventing their interaction with acetylated histones [53]. This disruption blocks BRD4’s binding to *MYC* super-enhancers, thereby reducing MYC expression [54]. The suppression of MYC’s activities by BET inhibitors (BETi) was reported in various types of tumors [55,56], and Fiorentino et al. [57] demonstrated that the therapeutic effects of BET inhibition include a decrease in MYC expression and suppression of its downstream targets, ultimately leading to inhibition of tumor cell proliferation. BET protein inhibitors can inhibit the expression or activity of MYC and MYCN in cancer cells. Additionally, it seems that MYC promotes RMS development by repressing the transcription of p21, a cyclin-dependent kinase inhibitor (CDKI) encoded by the *CDKN1A* gene. *p21* is a key regulator of the cell cycle, capable of halting progression at the G1 and S phases in response to various cellular stresses, including DNA damage and oncogene activation [58]. MYC suppresses p21 expression at the transcriptional level by directly binding to the promoter region of the *CDKN1A* gene and recruiting repressive cofactors [59]. These cofactors include the transcriptional repressor MIZ-1 (MYC-interacting zinc finger protein-1), which MYC antagonizes in a dose-dependent manner [60]. In the absence of MYC, MIZ-1 functions as an activator of p21 transcription, promoting cell cycle arrest. However, when MYC is overexpressed, it binds to MIZ-1 and converts it into a repressor by recruiting histone deacetylase (HDAC) complexes that modify chromatin to a repressive state [61]. The suppression of p21 by MYC is a critical event in the maintenance of uncontrolled proliferation, as it prevents p21 from inhibiting cyclin–CDK complexes, such as cyclin D-CDK4/6 and cyclin E-CDK2. This loss of p21-mediated regulation promotes unchecked cell cycle progression and is frequently observed in *MYC*-driven cancers [62]. Moreover, the inhibition of p21 contributes to the evasion of cellular senescence, a key barrier to tumorigenesis [63]. Therapeutically, targeting the MYC-p21 axis represents a promising strategy for cancer treatment. Small molecules or genetic approaches that disrupt MYC’s interaction with MIZ-1 or prevent the recruitment of repressive chromatin modifiers could restore p21 expression and reinstate cell cycle control. To give an example, Zhang et al. [29] found that treating RMS cells with an MYC inhibitor for 24, 48, and 72 h resulted in a significant time-dependent increase in p21 mRNA expression levels compared to untreated controls (Figure 9). Furthermore, apoptosis was significantly increased, while the cell cycle was not affected. This study revealed that *MYC* might be a suitable target for RMS clinical treatment.

## 4. MYC in Osteosarcoma

**Figure 10:**
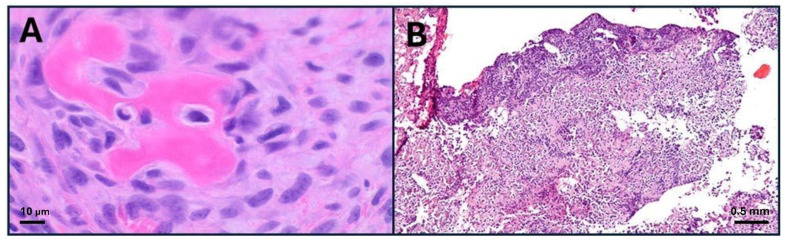
(**A**) Histopathology of osteosarcoma, showing tumor cells with high nuclear pleomorphism, but relatively less in cells entrapped in neoplastic bone matrix (appearing pink on this H&E-stained slide [66]; (**B**) small cell osteosarcoma, like other osteosarcomas, has osteoid, the extracellular organic component of bone. On H&E-stained sections, osteoid has a pink, homogeneous appearance and surrounds osteoblasts or osteosarcoma cells [66].

Osteosarcoma (OS) is the eighth most common cancer in children, accounting for 2.4% of all pediatric malignancies [64]. It typically affects the long bones (Figure 10) and is a highly aggressive tumor, often spreading to the lungs [65].

Preoperative, or neoadjuvant, chemotherapy represents one of the cornerstones of treatment. Its primary aim is to reduce the tumor mass to facilitate surgical intervention and increase the likelihood of achieving a complete resection. Additionally, it allows for an assessment of the tumor’s response to therapy, which serves as an important prognostic factor; the drugs most commonly used include Methotrexate, doxorubicin, and cisplatin [67].

Surgery remains the mainstay of treatment for achieving local disease control. Over the past few decades, the introduction of limb-sparing surgical techniques has significantly reduced the number of amputations, favoring limb-salvage procedures. These techniques involve tumor resection with clean margins, followed by bone reconstruction using metal prostheses, bone grafts, or biological implants [68]. With a combination of chemotherapy and surgery, the five-year survival rate for patients without metastatic disease at diagnosis is between 60% and 70% [69].

However, in cases where tumors are in particularly complex areas or when there is insufficient response to neoadjuvant chemotherapy, amputation may still be necessary.

Postoperative, or adjuvant, chemotherapy is crucial to eliminate any residual micro metastases, which are the primary cause of recurrence. The therapeutic regimen often mirrors that used in the neoadjuvant phase [70].

As mentioned earlier, pulmonary metastases are a common complication of OS. Surgical resection of metastases, when feasible, is associated with improved survival [71]. Furthermore, systemic chemotherapy continues to play a central role in the treatment of metastatic disease.

Genetic analysis of tumor cells from OS patients has revealed a loss of the *Retinoblastoma* gene (*RB*) in both copies (homozygous loss) and/or alterations in the *RB* gene product. *RB* produces a nuclear phosphoprotein (pRb) that plays a key role in suppressing cell cycle progression. Additionally, the *p53* tumor suppressor gene has been implicated in the development of OS. Mutations in this gene are frequently found in human carcinomas as well as in BS and STS. The *p53* gene encodes a nuclear phosphoprotein that functions as a transcription factor [72]. Furthermore, amplification or overexpression of the *MDM2* (Murine Double Minute 2) and *CDK4* genes has been observed in OS, suggesting their involvement in tumor development [73]. The *MDM2* gene encodes a protein that binds to and inactivates *p53*, while the CDK4 protein is a cyclin-dependent kinase that can phosphorylate and inactivate pRb (Table 2).

**Figure 11:**
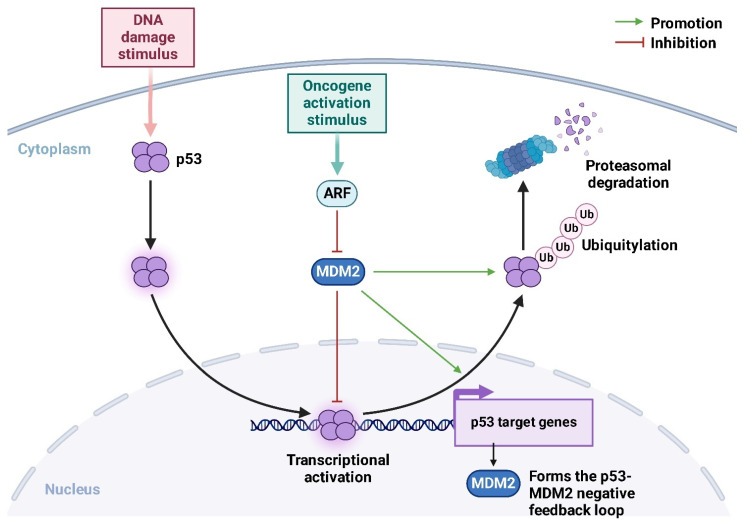
Under stress conditions, the p53 protein accumulates in the cell, binds in its tetrameric form to p53-response elements, and induces the transcription of genes involved in cell cycle control, apoptosis, DNA repair, differentiation, and senescence. However, in many cancers, including osteosarcoma (OS), MDM2 is often overexpressed or amplified, leading to unchecked inhibition of p53 and failure to induce cell cycle arrest or apoptosis in response to stress or DNA damage. Mechanistically, MDM2 stabilizes MYC mRNA by binding to AU-rich elements (AREs) within its 3′ untranslated region (UTR), preventing its degradation. This results in increased translation and accumulation of MYC protein, which in turn promotes uncontrolled proliferation and contributes to tumor development.

*MDM2* is a crucial oncogene encoding an E3 ubiquitin ligase that plays a central role in regulating the *p53* tumor suppressor pathway. By ubiquitinating p53, MDM2 targets it for proteasomal degradation, thus maintaining tight control over p53 activity [74]. This mechanism ensures that p53 levels remain low under normal physiological conditions, allowing for cell survival and proliferation (Figure 11) [75].

However, in many cancers, including OS, *MDM2* is overexpressed or amplified, leading to unchecked inhibition of p53 and a failure to induce cell cycle arrest or apoptosis in response to cellular stress or DNA damage [76]. Mechanistically, *MDM2* stabilizes *MYC* mRNA by binding to AU-rich elements (AREs) within its 3′ untranslated region (UTR). This interaction prevents *MYC* mRNA degradation, resulting in increased translation and accumulation of MYC protein. The stabilization of *MYC* enhances its ability to drive the transcription of genes that promote cellular growth, metabolic reprogramming, and the evasion of apoptosis [77]. At the same time, MYC directly transcriptionally activates *MDM2* by binding to its promoter, further increasing *MDM2* expression [78]. This reciprocal regulation creates a powerful oncogenic feedback loop that supports tumor growth and survival.

**Figure 12:**
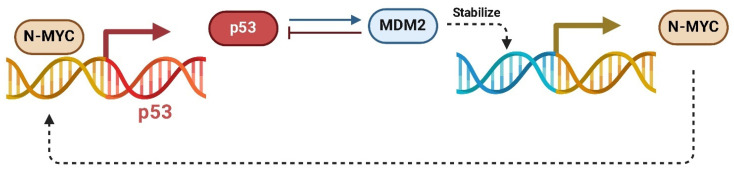
Feedback regulation in the *MYC*-*p53*-*MDM2* axis.

In the context of OS, this *MDM2*/*MYC* axis is particularly significant. OS is characterized by aggressive growth, a high metastatic potential, and frequent resistance to therapy. The combined effects of *MYC* and *MDM2* dysregulation contribute to this phenotype by enhancing cellular proliferation, evading apoptosis, and impairing the DNA damage response (Figure 12) [79].

Specifically, *MDM2*-mediated stabilization of *MYC* facilitates its ability to suppress key cell cycle inhibitors such as p21 and p27, tipping the balance in favor of uncontrolled proliferation [80]. Simultaneously, *MYC* represses pro-apoptotic factors and promotes the expression of anti-apoptotic proteins such as BCL-2, enabling tumor cells to survive under stress conditions, including hypoxia and treatment-induced DNA damage [81].

**Figure 13:**
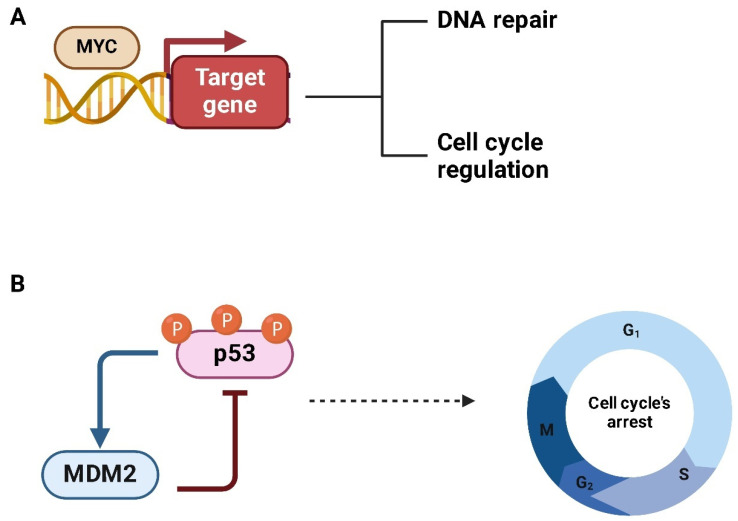
(**A**) MYC enhances the transcription of genes essential for DNA repair; (**B**) MDM2 concurrently suppresses p53-driven cell cycle checkpoints. This creates a paradoxical scenario where repair mechanisms are initiated but lack adequate regulatory control.

This interplay between MDM2 and MYC also influences TME [41]. By stabilizing MYC, MDM2 indirectly supports the upregulation of angiogenic factors such as VEGF [82], fostering the development of a robust vascular network that sustains tumor growth and facilitates invasion. Furthermore, the dysregulation of this axis enhances genomic instability [83]. While MYC promotes the transcription of genes involved in DNA repair (Figure 13A), MDM2 simultaneously inhibits p53-mediated cell cycle checkpoints (Figure 13B), leading to a paradoxical situation where repair processes are activated without sufficient oversight. This dual effect increases the adaptability of OS cells and their resistance to genotoxic therapies like radiation and chemotherapy.

Targeting the MDM2/MYC axis represents a promising therapeutic strategy for OS and other sarcomas, just as what happens in myeloma [79]. MDM2 inhibitors, such as Nutlins, disrupt the interaction between MDM2 and p53, leading to p53 reactivation and tumor cell apoptosis [84]. Importantly, these inhibitors also destabilize *MYC* mRNA by preventing its stabilization by MDM2, thereby reducing MYC protein levels [85]. This dual mechanism of action addresses two of the most critical drivers of OS progression. Preclinical studies have shown that combining MDM2 inhibitors with other therapeutic agents, such as chemotherapy or targeted inhibitors of MYC, can synergistically suppress tumor growth and overcome drug resistance [86].

**Figure 14:**
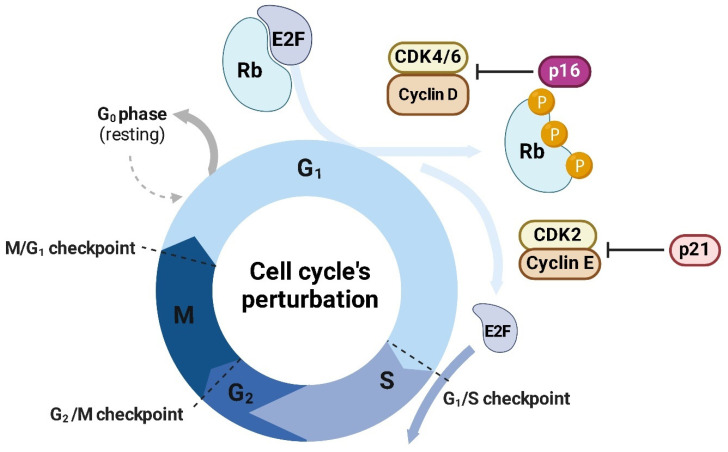
Mutations in cell cycle-related genes, such as *RB1* and *CDK4/6*, play a critical role in driving chemotherapy resistance in osteosarcoma. The cell cycle is tightly regulated by key proteins and checkpoints to ensure proper progression through its phases. For instance, the retinoblastoma protein (Rb) and *E2F* transcription factor control the G_1_/S checkpoint, preventing premature DNA replication. Cyclin D–CDK4/6 complexes phosphorylate Rb, releasing E2F and enabling cell cycle progression. Similarly, cyclin E–CDK2 complexes further reinforce this transition, driving cells into the S phase. Dysregulation of these pathways, such as overexpression of *CCNE1* or mutations in *RB1*, can lead to unchecked cell cycle progression and replication stress, contributing to cancer proliferation and resistance to therapy.

Cyclin-dependent kinase 4 (CDK4) is a serine/threonine kinase that plays a pivotal role in cell cycle regulation, particularly at the G1-to-S phase transition [87]. CDK4 forms an active complex with cyclin D (D1, D2, or D3), phosphorylating the retinoblastoma (RB1) protein. This phosphorylation leads to the release of *E2F* transcription factors, which in turn activate the transcription of genes required for DNA synthesis and cell cycle progression [88,89] (Figure 14). The CDK4/cyclin D axis is tightly regulated under normal physiological conditions, ensuring balanced cell proliferation and preventing unchecked division [90].

In many cancers, including OS, *CDK4* is frequently overexpressed or amplified, contributing to aberrant cell cycle progression [91]. Amplification of *CDK4*, often seen in sarcomas such as OS and liposarcoma, bypasses the need for normal growth signals, allowing tumor cells to proliferate uncontrollably [92]. This dysregulation also creates a permissive environment for genomic instability, further driving tumor progression.

The interplay between CDK4 and MYC forms a pivotal oncogenic axis that drives the progression of various cancers, including sarcomas [93]. This axis operates through reciprocal regulation, where each molecule amplifies the activity and stability of the other, fostering an environment conducive to uncontrolled proliferation, metabolic reprogramming, and survival.

**Figure 15:**
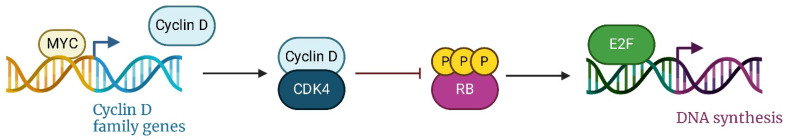
MYC acts as a key transcriptional regulator, directly stimulating the expression of genes encoding the cyclin D family (*CCND1*, *CCND2*, and *CCND3*). Cyclin D proteins are vital for activating CDK4, forming CDK4–cyclin D complexes that phosphorylate and deactivate the retinoblastoma protein (RB1). This phosphorylation prevents RB1 from binding and restraining E2F transcription factors, allowing E2Fs to promote the expression of genes required for DNA synthesis and entry into the S phase of the cell cycle.

MYC functions as a transcriptional master regulator and directly promotes the expression of genes encoding the cyclin D family (*CCND1*, *CCND2,* and *CCND3*) [94]. Cyclin D proteins are critical activators of *CDK4*, forming CDK4–cyclin D complexes that phosphorylate and inactivate the retinoblastoma protein (RB1) [95]. This phosphorylation disrupts RB1’s ability to sequester E2F transcription factors, liberating E2Fs to drive the transcription of genes essential for DNA synthesis and S-phase entry (Figure 15) [96].

This MYC-driven upregulation of *CDK4* activity ensures a rapid transition through the G1 phase of the cell cycle, bypassing growth-inhibitory signals. MYC’s role in enhancing *cyclin D* transcription creates a feedback loop in which MYC both induces and relies on CDK4 activity to maximize its oncogenic potential.

**Figure 16:**
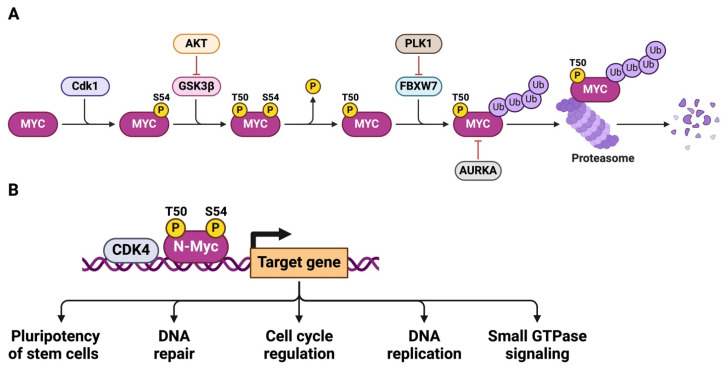
(**A**) Under normal conditions, FBXW7 functions as a tumor suppressor by targeting MYC for proteasomal degradation; (**B**) CDK4 enhances the stability of the MYC protein, protecting it from degradation and boosting its transcriptional activity. This is achieved by phosphorylating MYC at specific sites, which blocks the binding of E3 ubiquitin ligases like FBXW7.

In a complementary mechanism, CDK4 stabilizes MYC protein, preventing its degradation and amplifying its transcriptional activity [97]. CDK4 achieves this through phosphorylation of MYC at specific residues, which inhibits the recruitment of E3 ubiquitin ligases such as FBXW7 [98]. Normally, FBXW7 targets MYC for proteasomal degradation, acting as a tumor suppressor (Figure 16A) [99]. By inhibiting this degradation, CDK4 extends the half-life of MYC, allowing for sustained transcriptional activation of its target genes (Figure 16B).

Furthermore, CDK4 indirectly contributes to MYC stabilization by modulating chromatin accessibility [100]. Active CDK4–cyclin D complexes can phosphorylate chromatin regulators such as pRB and histone-modifying enzymes, creating a chromatin state that is more permissive for MYC binding. This enhanced chromatin recruitment increases the ability of MYC to amplify transcriptional programs that drive tumorigenesis.

A critical aspect of the CDK4-MYC axis is its self-reinforcing nature. CDK4 stabilizes MYC protein, which in turn drives the expression of cyclin D proteins and CDK4 itself, creating a powerful feedback loop. This loop ensures the sustained activity of both MYC and CDK4, even in the face of anti-proliferative signals.

The CDK4-MYC axis is particularly adept at overriding cellular checkpoints, including those mediated by tumor suppressors such as p16INK4a and p21 [101]. MYC represses the transcription of these cyclin-dependent kinase inhibitors [102] (CKIs), while CDK4 activity renders them functionally inert by phosphorylating and sequestering their target, RB1 [103]. This coordinated suppression of cell cycle inhibitors enables continuous cell cycle progression, a hallmark of cancer.

The CDK4-MYC axis is not limited to cell cycle regulation; it also plays a key role in the metabolic reprogramming of cancer cells [104]. MYC drives the expression of genes involved in glycolysis, glutaminolysis, and mitochondrial biogenesis, ensuring a steady supply of energy and biosynthetic precursors to fuel rapid proliferation [105]. CDK4 enhances this metabolic shift by stabilizing MYC, prolonging its activity as a transcriptional regulator of metabolic genes.

In addition, CDK4 indirectly supports metabolic adaptation by modulating the activity of enzymes involved in lipid synthesis and nucleotide biosynthesis, pathways that are frequently co-opted by MYC to support tumor growth [106].

Given its central role in OS biology, CDK4 represents an attractive therapeutic target.

In sarcomas like OS, CDK4/6 inhibitors such as Palbociclib, Ribociclib, and Abemaciclib have been developed to target CDK4/6 activity [107]. By inhibiting CDK4, these agents prevent the phosphorylation of RB1, halting cell cycle progression and disrupting the CDK4-MYC feedback loop [108]. These inhibitors prevent CDK4 from phosphorylating RB1, thereby halting cell cycle progression at the G1 phase [109]. This inhibition not only impairs proliferation but also destabilizes MYC protein, reducing its oncogenic influence. Combining CDK4/6 inhibitors with MYC-targeted therapies or standard chemotherapy has shown synergistic effects. For instance, CDK4 inhibition sensitizes tumor cells to DNA-damaging agents by restoring cell cycle checkpoints and allowing more effective engagement of apoptotic pathways [110].

The dependency of MYC-driven cancers on CDK4 presents opportunities also for synthetic lethality. Tumors that overexpress *MYC* often exhibit heightened sensitivity to CDK4/6 inhibition, making this axis a selective vulnerability in *MYC*-driven cancers.

The treatment of OS requires a multidisciplinary approach that combines surgery, chemotherapy, and, in selected cases, radiotherapy [111].

Despite advances in multidisciplinary treatment, the prognosis for metastatic OS remains poor, with a 5-year survival rate of less than 30% [112].

Given the current difficulty in identifying valid molecular targets for effective OS treatment and the demonstrated overexpression and amplification of *MYC* in this type of cancer, it would be worthwhile to investigate the effects of an MYC inhibitor to potentially establish the foundation for future therapeutic approaches.

## 5. MYC in Synovial Sarcoma

Synovial sarcoma (SyS) is a rare type of STS that primarily affects adolescents and young adults, accounting for approximately 5–10% of all STSs [113]. A widely accepted cause of SyS is a chromosomal abnormality known as T(x; 18) (p11.2; q11.2), which results in the formation of *SS18-SSX* fusion oncogenes. This chromosomal anomaly is found in more than 90% of SyS cases, leading to the belief that it plays a key role in the development of the disease [114]. SyS is mostly found in the lower limbs, particularly in the thigh area. This type of tumor is generally classified as high-grade and is associated with a poor prognosis [115]. In SyS, factors such as the size and depth of the tumor, its location, and the possibility of achieving complete surgical resection all play a significant role in determining the prognosis [116].

The standard treatment usually involves the surgical removal of the tumor with wide margins, often followed by chemotherapy or a combination of therapies [117].

Managing patients with rare subtypes can be challenging, as there is often a lack of extensive data from clinical trials and outcome studies to inform evidence-based decisions.

Similarly, data on clinical outcomes for SyS are limited, largely due to its low incidence and the difficulty of gathering studies with large sample sizes. Surgery remains the primary treatment for SySs. The chances of survival significantly increase when the entire tumor is successfully removed, with no signs of cancer elsewhere in the body. The success of the procedure largely depends on the tumor’s size and location. In some cases, radiation therapy may be administered before or after surgery to eliminate cancer cells, while chemotherapy is considered if the tumor cannot be completely removed or if the cancer has spread [118]. In recent years, there has been a rise in subtype-specific trials for sarcomas, reflecting a growing recognition of the considerable diversity in clinical behavior, biology, and genetic profiles among various STS subtypes. This shift also highlights recent advancements in tumor-specific therapies. Numerous targeted therapies have been evaluated for efficacy in SyS. These include agents targeting receptor tyrosine kinases (RTKs), epigenetic regulators, and immunomodulators.

**Figure 17:**
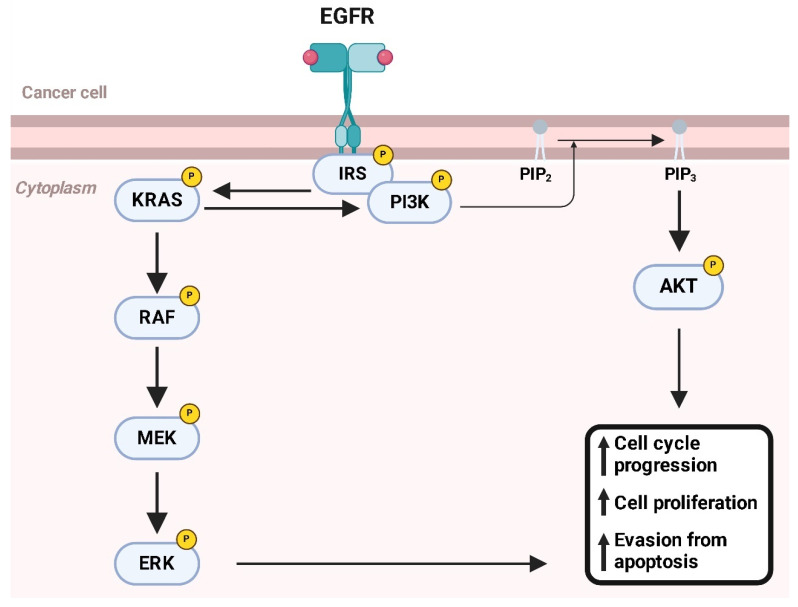
When the Epidermal Growth Factor Receptor (EGFR) is overexpressed, its activation promotes cell proliferation and resistance to apoptosis by signaling through the RAS/RAF/MEK and PI3K/AKT pathways.

RTKs are a large family of proteins that act as molecular switches, regulating numerous physiological processes [119]. These membrane-bound receptors transduce extracellular signals into intracellular responses through tyrosine phosphorylation mechanisms. RTKs are composed of three main domains: an extracellular domain that binds ligands such as growth factors (e.g., VEGFR, EGFR, or PDGFR) [120]; a transmembrane domain anchoring the receptor to the membrane [121]; and an intracellular domain with tyrosine kinase activity [122]. Upon ligand binding, the extracellular domain induces receptor dimerization, activating the intracellular kinase domain. This activation triggers phosphorylation of tyrosine residues on the receptor itself (autophosphorylation) and on downstream effector proteins [123]. These phosphorylation events create docking sites for signaling proteins, activating critical intracellular pathways such as PI3K/AKT, which promotes cell survival and growth [124]; RAS/RAF/MEK/ERK, which stimulates proliferation and differentiation [125]; and JAK/STAT, which regulates inflammation and hematopoiesis [126]. RTKs play essential roles in various physiological contexts, including embryonic development, angiogenesis, tissue repair, and metabolism. However, in SyS, these receptors are often upregulated, contributing to tumor progression [127]. Although SyS is primarily characterized by the chromosomal translocation t(X;18) (p11;q11), which generates the *SS18-SSX* gene fusion, RTKs play a significant role in its progression by driving dysregulated signaling pathways [128]. Several RTKs are overexpressed or hyperactivated in SyS, promoting its aggressive behavior. For example, EGFR (Epidermal Growth Factor Receptor) is overexpressed in some cases of SyS, and its activation drives proliferation and resistance to apoptosis via the RAS/RAF/MEK and PI3K/AKT pathways (Figure 17) [129].

The SS18-SSX fusion, a hallmark of SyS, alters gene expression and can enhance RTK activation [130]; this suggests that RTKs not only contribute independently to tumor biology but also interact with genetic alterations to drive malignancy [131].

In tumor cells, RTKs and MYC cooperate to promote a malignant phenotype [132]: aberrant activation of RTKs, through overexpression or activating mutations, increases the transcriptional expression of MYC by continuously stimulating the RAS/RAF/MEK and PI3K/AKT pathways [133]. At the same time, hyperactivation of RTKs blocks the degradation of MYC, prolonging its half-life and enhancing its oncotranscriptional activity [134]. This occurs through AKT-mediated phosphorylation of MYC, which makes it less susceptible to ubiquitin–proteasomal degradation [36]. Once stabilized and highly expressed, MYC acts as a “master regulator” of transcription, activating genes involved in proliferation, aerobic metabolism, angiogenesis, and tumor invasiveness [135]. Furthermore, MYC suppresses genes that regulate apoptosis, thus promoting cell survival.

In SyS, the interplay between MYC and receptor tyrosine kinases (RTKs), such as PDGFR and VEGFR, plays a critical role in tumor progression, highlighting a complex relationship that drives oncogenesis [136]. MYC, a well-known proto-oncogene, acts as a transcriptional amplifier regulating genes involved in cell proliferation, metabolism, and survival. In SyS, *MYC* is often overexpressed, contributing to aggressive tumor behavior [137]. Similarly, RTKs like PDGFR and VEGFR promote tumor growth and angiogenesis through downstream signaling pathways such as PI3K/AKT and RAS/RAF/MEK [138].

**Figure 18:**
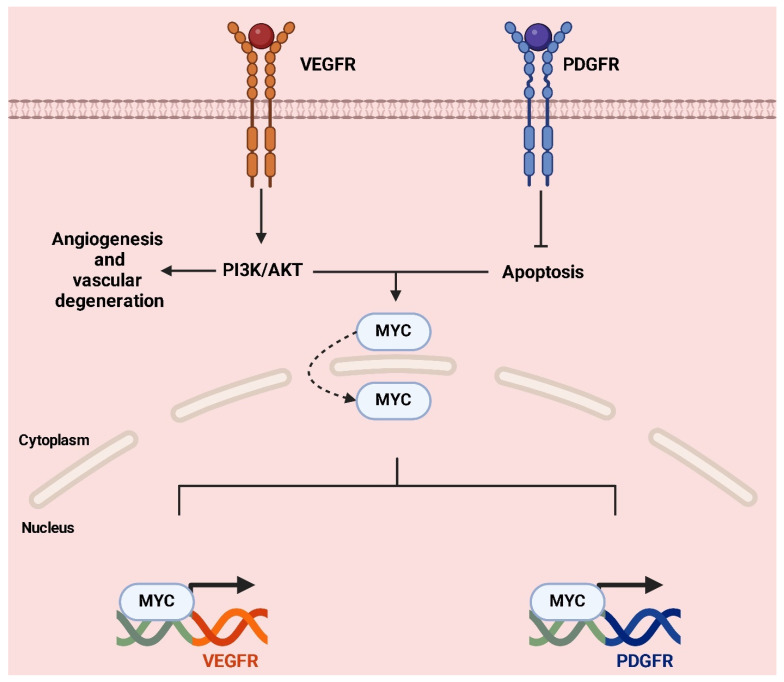
In SyS, the interaction between MYC and RTKs, such as PDGFR and VEGFR, drives tumor progression by promoting cell proliferation, metabolism, and angiogenesis. Overexpressed MYC amplifies growth signals, while RTKs sustain MYC activity through positive feedback loops.

The activation of RTKs can upregulate MYC expression by stabilizing the protein and promoting its nuclear localization through signaling cascades. This amplification creates a feedback loop where MYC, in turn, enhances the expression of ligands like VEGF, which bind to RTKs and sustain their activation. This cycle ensures continuous proliferative and survival signals within tumor cells, enabling unchecked growth (Figure 18) [139].

Furthermore, MYC and RTKs jointly influence cellular metabolism. MYC drives glycolysis and lipid biosynthesis, while RTKs facilitate nutrient uptake and energy production, ensuring that the tumor has a steady supply of resources to support rapid proliferation [140].

A significant aspect of this interplay lies in the regulation of angiogenesis. VEGFR is a key player in vascular development, promoting endothelial cell migration, proliferation, and survival [141]. In SyS, VEGFR signaling is often enhanced by increased levels of VEGF, which is partly driven by MYC. Beyond this, MYC further amplifies angiogenesis by regulating hypoxia-induced factors like HIF-1α and angiogenic genes such as *VEGF-A* and *ANGPT2* [142]. Together, MYC and RTKs foster the formation of new blood vessels that supply nutrients and oxygen to the tumor. However, the resulting vasculature is often abnormal, characterized by immature and leaky vessels. This dysfunctional network contributes to a hypoxic tumor microenvironment that selects for more aggressive tumor phenotypes and drives resistance to therapies [143].

The MYC-RTKs axis also shields tumor cells from apoptosis and senescence. MYC’s ability to suppress senescence, combined with the pro-survival signaling of RTKs, creates a robust defense against cell death, ensuring the survival of even highly stressed tumor cells. This dual contribution makes the interplay between MYC and RTKs not only essential for tumor maintenance but also a significant obstacle to effective treatment [144].

Targeting this dual axis offers promising therapeutic opportunities. Inhibitors of RTKs, such as Pazopanib or Sunitinib, are already being used to block angiogenesis and disrupt tumor growth [145]. However, because MYC operates downstream and amplifies the effects of RTK signaling, combining RTK inhibitors with strategies to suppress MYC activity could enhance therapeutic efficacy. Approaches that indirectly target MYC, such as epigenetic modulators or inhibitors of its cofactors, could complement RTK-targeted therapies by disrupting the synergistic feedback loop and limiting tumor adaptability.

Among epigenetic therapies, the EZH2 inhibitor Tazemetostat has garnered the most attention in clinical studies for SyS. Tazemetostat shows increased activity in tumors lacking integrase interactor 1 (INI1, also known as SMARCB1), as INI1 loss allows EZH2 to act as an oncogenic driver in tumor cells. Further studies are currently underway to evaluate the efficacy of Tazemetostat in SyS [146]. However, despite encouraging preclinical and early-stage results, most of these approaches have struggled to achieve lasting success in clinical settings, generally offering only short-lived benefits to a small subset of SyS patients. Identifying an effective molecular target to combat SyS remains challenging and, as demonstrated by Somers et al. [147], SyS shows high levels of *MYC*, and it became clear that *SS18-SSX* likely targets distinct sets of genes that are associated with specific biological processes like *MYC* [148].

Given its central role and interactions with several key pathways in SyS, the overexpression of *MYC* in this tumor type highlights it as a potential target worth exploring for the development of new therapies aimed at slowing SyS progression.

## 6. MYC in Malignant Rhabdoid Tumor (MRT)

Malignant Rhabdoid Tumors (MRTs) account for less than 5% of malignant liver tumors in infants. These tumors are characterized by aggressive clinical behavior, resistance to chemotherapy and radiation, and a high mortality rate [149]. At diagnosis, patients typically present with symptoms such as fever, abdominal distension, markedly elevated lactate dehydrogenase (LDH) levels, and either normal or reduced alpha-fetoprotein (AFP) levels [150]. One of the biggest challenges in treating MRT is achieving an accurate diagnosis, particularly in distinguishing it from small cell undifferentiated hepatoblastoma (SCUD-HB), which requires a different therapeutic approach [151]. While hepatoblastoma tipically affects children within the first 18 months of life, MRT primarily occurs in infants younger than 8 months [152].

Currently, there is no standardized treatment protocol for MRT. Most patients receive intensive multimodal therapies, which typically involve the early surgical resection of the primary tumor if a gross total resection (GTR) is possible. This is often accompanied by intensive multidrug chemotherapy regimens, and localized radiotherapy targeting all disease sites. In some cases, high-dose chemotherapy (HDCT) followed by autologous stem-cell rescue is also employed. Common cytostatic agents used across these approaches include anthracyclines, alkylating agents, platinum-based compounds, and vinca alkaloids [153].

From a molecular point of view, as demonstrated by Gadd et al. [154], *SMARCB1* ranked among the top five most significantly differentially expressed genes in MRT. Moreover, 12 of the top 114 genes identified were previously shown to express *SMARCB1* in earlier gene expression studies. This group includes genes that exhibit concordant differential expression following the induction of *SMARCB1* in MRT cell lines.

**Figure 19:**
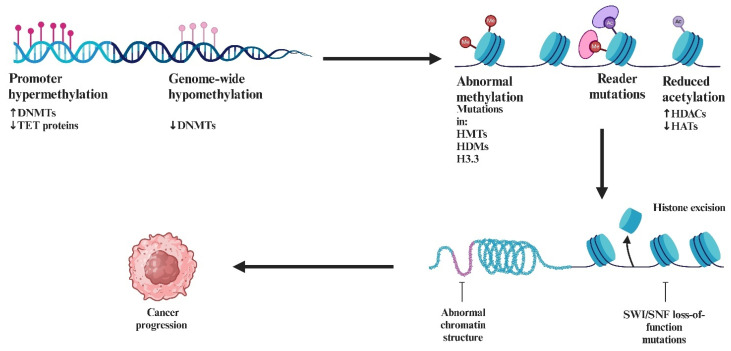
The SWI/SNF complex plays a crucial role in the regulation of epigenetic changes, acting as a central player in chromatin remodeling. This multi-protein complex is responsible for ejecting or restructuring nucleosomes, thereby controlling the accessibility of transcription factors to DNA. Its role is fundamental in maintaining normal gene expression patterns and cellular differentiation.

*SMARCB1* plays a crucial role in the pathogenesis of MRTs, a rare and aggressive form of pediatric cancer [155]. The loss of *SMARCB1* is considered the central event in the development of these tumors, and it is often caused by biallelic inactivation of the *SMARCB1* gene through mutations or deletions [156]. As a core subunit of the *SWI/SNF* chromatin remodeling complex, *SMARCB1* is essential for the regulation of gene expression, chromatin structure, and cellular differentiation. Its loss leads to significant transcriptional dysregulation, preventing normal cellular differentiation and promoting uncontrolled cell proliferation [157]. The absence of *SMARCB1* destabilizes the *SWI/SNF* complex, which is involved in altering nucleosome positioning to regulate gene accessibility and transcription [158]. This disruption in chromatin remodeling results in epigenetic changes that silence tumor suppressor genes and activate oncogenic pathways. This defect contributes to the aggressive nature of MRTs, where cells fail to undergo senescence or differentiation, and, instead, continue to proliferate abnormally (Figure 19) [159].

In MRTs, the loss of *SMARCB1* is not only a marker of tumor progression but also a driving force behind the cellular behaviors that define these cancers, including abnormal growth, resistance to apoptosis, and failure to differentiate.

The loss of *SMARCB1* destabilizes the SWI/SNF complex, leading to epigenetic dysregulation that can impact various signaling pathways, including MYC.

Research has shown that *SMARCB1* physically interacts with MYC, regulating its oncogenic activity. Under normal conditions, SMARCB1 acts as a brake on MYC, limiting its ability to activate genes involved in cell proliferation and survival [160].

**Figure 20:**
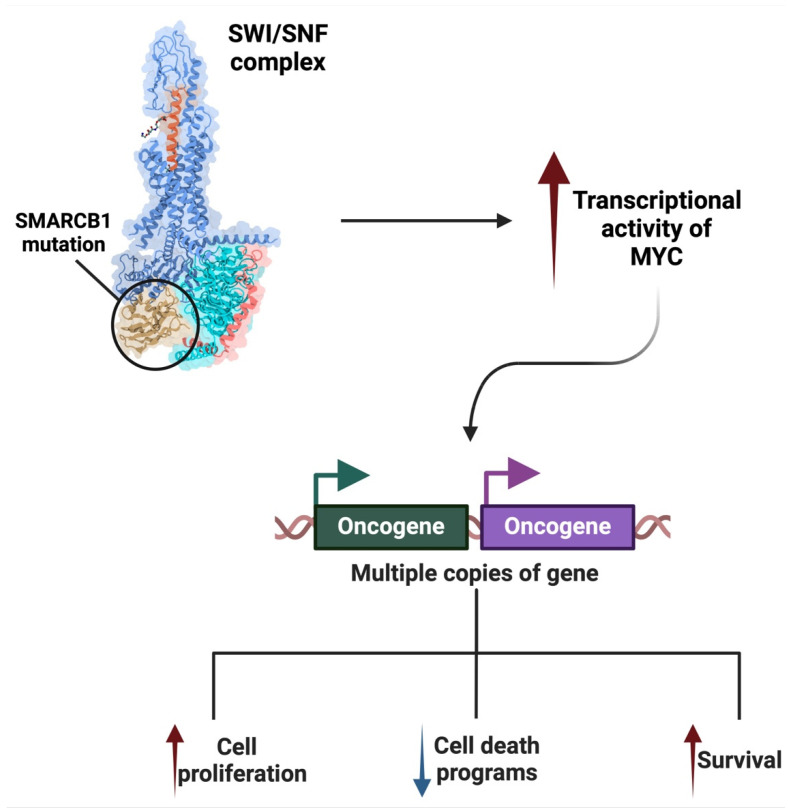
When *SMARCB1* is lost or mutated, its ability to regulate *MYC* is eliminated. As a result, MYC’s transcriptional activity increases, driving the overexpression of oncogenic genes that promote cell proliferation, improve survival, and inhibit programmed cell death mechanisms.

When *SMARCB1* is lost or mutated, as seen in malignancies like MRTs, the regulatory control exerted by SMARCB1 over MYC is abolished [161]. This results in increased transcriptional activity of MYC, leading to the overexpression of oncogenic genes that drives cell proliferation, enhances survival, and suppresses programmed cell death mechanisms (Figure 20). Consequently, cells acquire a more aggressive phenotype, characterized by unchecked growth and heightened resistance to therapies [162].

The loss of *SMARCB1* also induces significant epigenetic changes. In its absence, the SWI/SNF complex can no longer effectively remodel chromatin, creating a transcriptionally permissive environment for *MYC* [161]. This not only amplifies MYC’s activity but also facilitates its cooperation with other oncogenic pathways. The increased expression of MYC target genes, coupled with the activation of survival pathways such as PI3K/AKT, further drives oncogenesis [163].

This dependency has significant therapeutic implications: studies have demonstrated that SWI/SNF-deficient cancers, including MRT, are particularly sensitive to MYC inhibition [164]. Targeting MYC directly, or its downstream pathways, has shown promise in preclinical models, suggesting a potential avenue for treatment in this otherwise intractable malignancy.

Given the current lack of effective therapy for the treatment of MRT, coupled with evidence of *MYC* overexpression in these tumors, it would be worthwhile to explore this avenue further. Initiating a series of studies to investigate the potential impact of an MYC inhibitor could offer valuable insights, potentially halting or even reversing tumor progression.

## 7. MYC in Leiomyosarcoma

**Figure 21:**
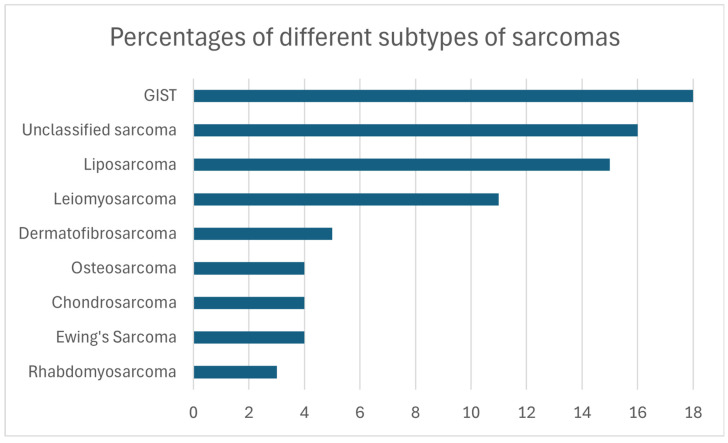
Incidence rates of the main histological subtypes of sarcoma.

Leiomyosarcoma (LMS) is among the most common STSs, accounting for an estimated 10% of all newly diagnosed cases (Figure 21). It represents a significant proportion of soft tissue and abdominal–pelvic sarcomas, second only to liposarcomas, and is the most prevalent sarcoma originating from large blood vessels [165].

Similar to other STSs, the overall incidence of LMS increases with age, peaking in the seventh decade of life. In contrast, uterine LMS can occur from the third decade onward but is most frequently diagnosed in the perimenopausal age group, typically during the fifth decade. The sex distribution of LMS varies based on tumor location. Women are more commonly affected by retroperitoneal and inferior vena cava LMSs, whereas noncutaneous soft tissue and cutaneous LMSs show a slight male predominance [166].

**Figure 22:**
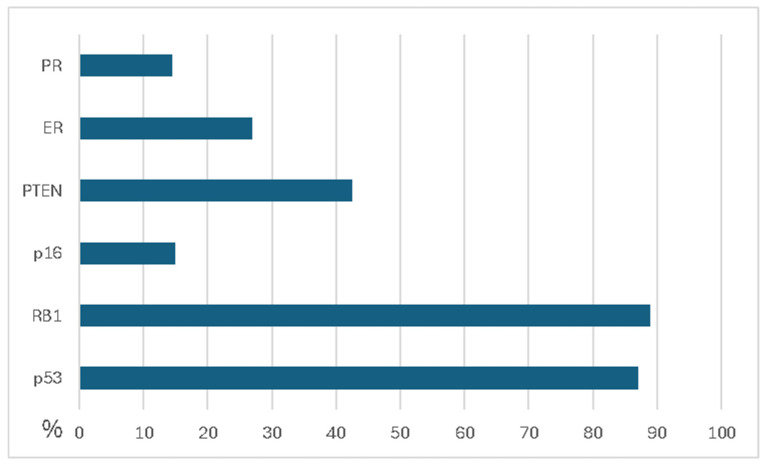
Frequent gene aberrations in LMS, elaborated from [167].

LMS is often associated with aggressive tumor biology and a higher risk of developing distant metastatic disease compared to most other sarcoma subtypes. The prognosis for patients with uterine LMS is particularly poor, underscoring the urgent need for more effective treatment options. Genetically, LMS exhibits a complex karyotype and is characterized by a low tumor mutational burden. Common genetic alterations include mutations in *p53*, *RB1*, and *PTEN*, as well as disruptions in DNA damage response pathways (Figure 22).

**Figure 23:**
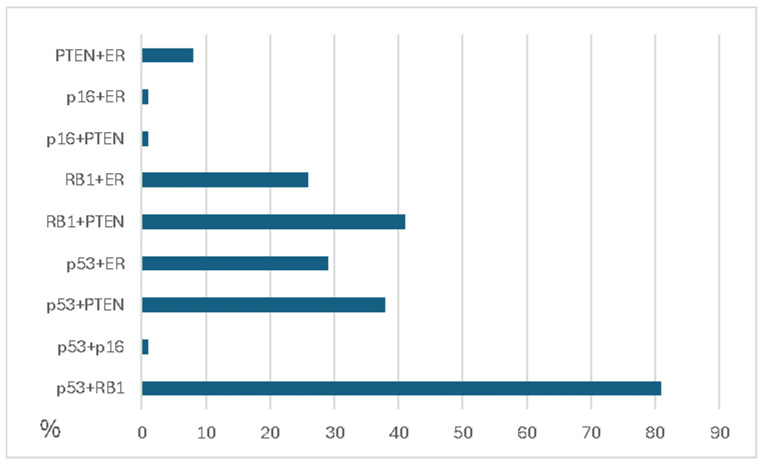
Associations of *p53*, *RB1*, *p16*, and *PTEN* aberrations and moderate–strong ER expression in LMS, elaborated from [167].

These factors may contribute to the tumor’s resistance to immune checkpoint inhibitor monotherapy [168] because these aberrations could also be present in tandem, leading to a more aggressive phenotype (Figure 23).

A pretreatment biopsy is essential, with core biopsy being the preferred method. This approach minimizes tissue disruption while providing sufficient material for a thorough pathological evaluation [169]. Surgical resection remains the primary treatment for localized LMS, regardless of its site of origin [170]. The aim of surgical resection is to achieve complete tumor removal with negative microscopic margins. Overall, LMS is among the more chemosensitive types of STS. Neoadjuvant chemotherapy with doxorubicin is recommended for large (>5 cm), intermediate-to-high-grade LMSs of the extremities and uterus [171] and is currently being investigated for retroperitoneal LMSs [172].

**Figure 24:**
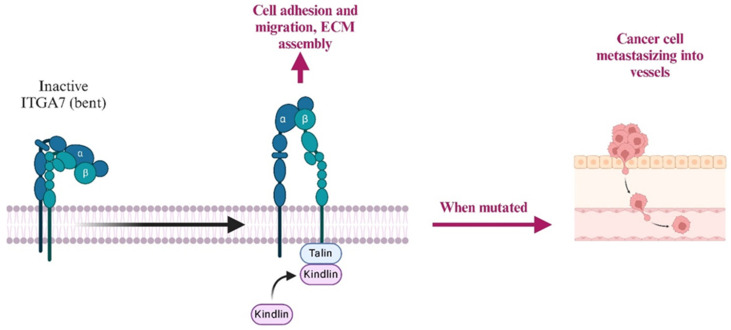
Integrin alpha-7 (ITGA7) is primarily known for its involvement in interactions between cells and the extracellular matrix (ECM). ITGA7 activation leads to cell adhesion, migration, and signaling, making it a key player in cancer progression and metastasis. In the context of LMS, ITGA7’s function becomes particularly relevant because it promotes metastasis by enhancing the adhesion and migration of cancer cells to distant sites. This occurs through its activation of downstream signaling pathways.

Integrin alpha-7 (ITGA7) is a transmembrane receptor that mediates cell–extracellular matrix (ECM) interactions, primarily binding to laminin in the basement membrane. This interaction is crucial for maintaining the structural integrity and function of smooth muscle tissues. In LMS, alterations in ITGA7 expression can disrupt these cell–ECM interactions, facilitating tumor cell detachment, invasion, and metastasis (Figure 24) [173]. Studies have shown that the downregulation or mutation of *ITGA7* is associated with increased tumor aggressiveness and poor prognosis in various cancers, including LMS [173].

The expression levels of ITGA7 have been found to vary across different tumor types. The overexpression of ITGA7 correlates with worse overall survival in certain cancers, while downregulation is associated with a poor prognosis in others. In LMS, the specific pattern of ITGA7 expression and its prognostic implications require further investigation to fully elucidate its role in tumor progression [174]. ITGA7 also plays a role in intracellular signaling pathways that regulate cell survival, proliferation, and apoptosis. It has been shown to interact with high-temperature requirement A2 (HtrA2), a serine protease involved in apoptotic processes. The interaction between ITGA7 and HtrA2 can induce apoptosis in cancer cells, suggesting a tumor-suppressive function. Loss or mutation of ITGA7 may impair this apoptotic pathway, allowing cancer cells to evade programmed cell death and contributing to tumor progression [175].

Immunohistochemical studies have frequently identified overexpression of the proto-oncogene *MYC* in uterine smooth muscle tumors, including LMS. In an analysis of 23 LMS cases, *MYC* overexpression was observed, suggesting its involvement in the tumorigenesis of these malignancies [176]. The overexpression of *MYC* has been linked to a poor prognosis across various cancer types. In a study examining MYC expression in 28 cases of deep soft tissue LMS, a significant correlation was found between MYC expression and both metastasis-free survival and overall survival. Patients with high MYC expression had worse outcomes compared to those with low expression, indicating that *MYC* could serve as a valuable prognostic marker in LMS [177]. MYC also plays a pivotal role in cellular transformation by activating specific transcription factors. In several sarcoma subtypes, MYC upregulates the transcription factor TBX3, which in turn promotes proliferation, tumor formation, migration, and invasion. This evidence suggests that the MYC/TBX3 axis may contribute to malignant transformation in LMS [178].

Although no direct studies have explored the interaction between ITGA7 and MYC in LMS, it is plausible to hypothesize that the loss of ITGA7 may contribute to the dysregulation of signaling pathways that enhance MYC’s oncogenic activity. While direct studies on the interaction between ITGA7 and MYC in LMS are lacking, some experimental data have implied that the oncoprotein MYC is a direct repressor of ITGA7 gene transcription [179], and research in other cancer types suggests that ITGA7 or other integrins can modulate intracellular signaling pathways that may intersect with MYC’s functions [180].

For example, in colorectal cancer (CRC), MYC plays a pivotal role as a transcription factor, regulating the expression of numerous genes involved in cell proliferation, differentiation, and survival. Among its downstream targets, integrins stand out for their crucial involvement in tumor progression, particularly in cell adhesion, migration, and invasion. Integrins, as heterodimeric receptors, mediate interactions between cells and the ECM, influencing key processes such as tumor angiogenesis and metastasis [181].

MYC is known to modulate the expression of specific integrins, thereby impacting CRC behavior. For example, studies have shown that MYC can upregulate integrin β1 (ITGB1), which enhances the attachment of cancer cells to the ECM and facilitates their invasive potential. Elevated levels of ITGB1 are frequently associated with more aggressive tumor phenotypes and poorer clinical outcomes in CRC patients [182].

Moreover, MYC-driven integrin expression affects the activation of downstream signaling pathways, such as the focal adhesion kinase (FAK) and SRC kinase pathways. These pathways promote cytoskeletal remodeling, increased motility, and resistance to apoptosis, hallmarks of cancer metastasis (as ITGA7). MYC’s influence on integrin expression creates a feedback loop in which integrin-mediated signaling enhances MYC activity, further exacerbating tumor progression [183].

Interestingly, MYC also interacts with integrin αV (ITGAV), another critical player in different types of cancer [184]. ITGAV is involved in angiogenesis and metastatic spread by modulating the tumor microenvironment. Studies suggest that MYC indirectly enhances ITGAV-mediated pathways, contributing to the establishment of pre-metastatic niches and supporting the survival of circulating tumor cells [185].

In addition to these mechanisms, MYC’s regulation of integrins may confer therapeutic resistance. Tumors with high MYC activity often exhibit resistance to conventional therapies, partly due to enhanced cell–ECM interactions mediated by integrins [183]. This highlights the importance of targeting the MYC/integrin axis as a potential therapeutic strategy.

The insights gained from CRC studies provide a valuable framework for investigating the potential interactions between MYC and integrins in LMS. Given the shared mechanisms of tumor progression across cancer types, exploring whether MYC regulates integrin pathways in LMS could uncover novel targets for therapeutic intervention.

## 8. MYC in Ewing’s Sarcoma

**Figure 25:**
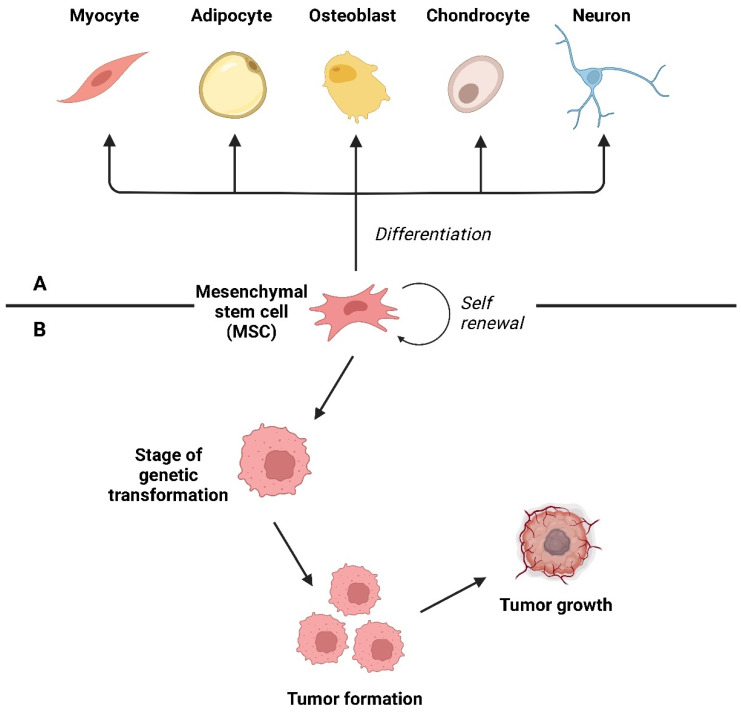
(**A**) Under physiological conditions, the mesenchymal stem cell retains its dual ability to self-renew and differentiate into various tissue-specific cells; (**B**) under the genetic pressure of oncogenic mutations, the mesenchymal stem cell embarks on a path of specialization into a tumor cell, driving tumor onset and progression.

Ewing’s sarcoma (ES) is an aggressive tumor primarily affecting adolescents and young adults, accounting for 10% to 15% of all bone sarcomas. First described by James Ewing in 1921, this condition encompasses classic ES of bone, extra-skeletal ES, the malignant small cell tumor of the chest wall (Askin tumor), and soft tissue-based primitive neuroectodermal tumors. Despite their different locations, these sarcomas share similar histologic and immunohistochemical features, suggesting they arise from mesenchymal progenitor cells (Figure 25) [186].

ES is the second most common primary bone malignancy in adolescents and young adults with a median age of 15 years and accounts for less than 5% of all STSs. ES in most cases occur between the ages of 10 and 15, with approximately 30% of cases diagnosed in children under 10 and another 30% in adults over 20. The condition shows a male predominance, with a male-to-female ratio of 3 to 1. It is more frequently seen in White individuals compared to Black, Asian, Hispanic, or African populations, though the reasons behind this racial disparity have not yet been fully explored.

**Figure 26:**
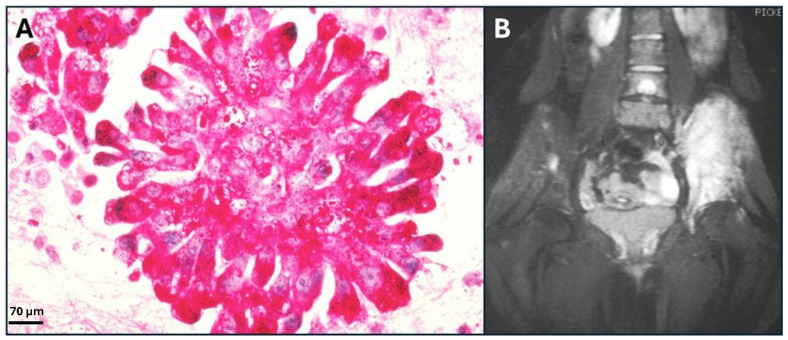
(**A**) Ewing’s sarcoma metastasized to the pleural fluid. Stained PAS for glycogen [188]; (**B**) magnetic resonance imaging (MRI) image of human skeleton. MRI diagnoses Ewing’s sarcoma of the right hip [189].

ES can arise in various locations, but it is most observed in long bones, particularly in the femoral diaphysis, as well as in the tibia and humerus, which are also frequent sites, although one of the earliest and most common secondary sites for metastasis is the pulmonary tissue (Figure 26A). It is also commonly located in the pelvic bones, such as the iliac bone, especially in adolescent patients [187] (Figure 26B).

The true incidence of ES in older populations remains unknown [190]. ES’s family tumors are defined by non-random chromosomal translocations that result in fusion genes, which produce abnormal transcription factors. The t(11;22)(q24;q12) translocation is found in 85% of cases and leads to the formation of the EWS-FLI-1 fusion protein. In contrast, the t(21;12)(22;12) translocation and other rarer translocations result in the *EWS-ERG* fusion, accounting for the remaining 10% to 15% of cases.

The standard of care for patients with or without metastasis includes interprofessional treatment with chemotherapy and local therapy, including surgery and radiotherapy. Broadly, systemic therapy is the cornerstone of treatment for all ES patients; in the United States, this consists of VDC (vincristine/doxorubicin/cyclophosphamide) with alternating IE (ifosfamide/etoposide). After induction chemotherapy, clinicians typically recommend local therapy using radiation, surgery, or a combination of both. Nonmetastatic disease has a 5-year survival rate of 75% to 80%, while that of metastatic disease is around 30% [191]. Radiotherapy is used for patients with ES who cannot obtain negative margins or have inoperable or metastatic disease. While there have been no direct prospective trials comparing radiotherapy to surgical resection, retrospective data suggest local failure rates are better with surgery (4% vs. 15%), and local failure appeared higher in those >18 years with pelvic tumors [192]. In patients who undergo both surgery and radiotherapy, the local failure rate is 6.6%. No significant difference in survival has been observed between treatment with surgery alone, radiotherapy alone, or the combination of both [193]. Radiotherapy is also used in metastatic settings to control the disease and may help improve event-free survival [194].

Despite therapeutic advances in ES over the past decade that have improved the 5-year survival rate from 10% to 55–60%, one-quarter of cases are diagnosed with distant metastases, reducing the 5-year survival rate to 15–30% [195]. In general, ES is difficult to treat. Patients have frequent relapses and require complex treatment regimens, including surgery, radiotherapy, and chemotherapy [196]. The complexity of ES treatment, combined with its limited effectiveness, highlights the need for new therapeutic strategies. While the development of new drugs, active molecules, and combination therapies is crucial, another key aspect often overlooked is the effective delivery of these substances to the target cells. Precise drug delivery systems are essential to minimize the side effects commonly seen with non-specific chemotherapy treatments, while also enhancing therapeutic efficacy [197].

**Figure 27:**
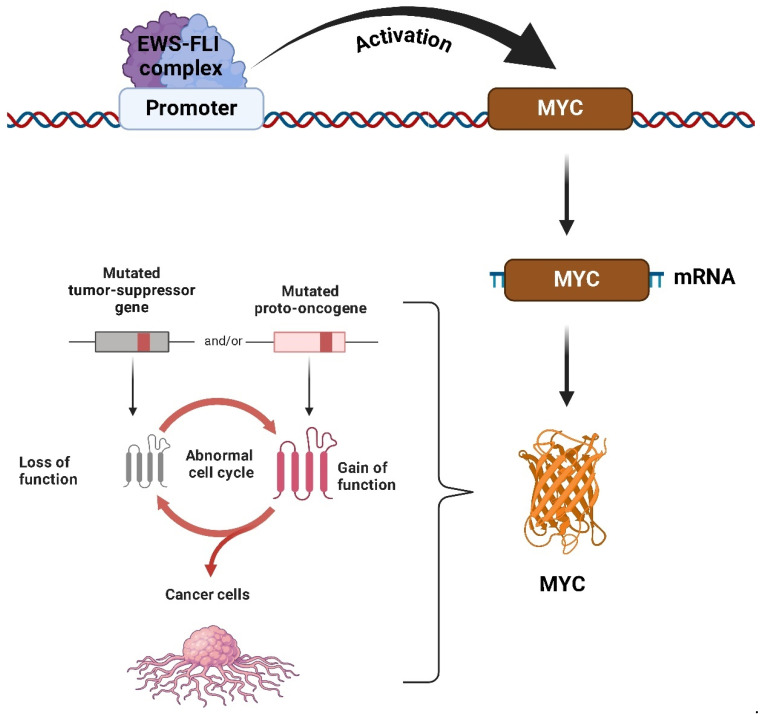
The overexpression of *MYC* in Ewing’s sarcoma (ES) is mainly driven by the fusion protein EWS-FLI-1, a key characteristic of the disease. EWS-FLI-1 directly binds to the promoter region of the MYC gene, boosting its transcription. This leads to elevated MYC levels, which further amplify the oncogenic processes initiated by EWS-FLI-1, creating a crucial pathway in the development of ES.

Sollazzo et al. [198] have reported that the *MYC* gene is overexpressed in approximately 50% of ES cases, a phenomenon closely associated with increased tumor aggressiveness and poor clinical outcomes. This overexpression is largely driven by the fusion protein EWS-FLI-1, a hallmark of ES, which directly binds to the promoter region of the MYC gene and enhances its transcriptional activation (Figure 27). The resulting elevated levels of *MYC* amplify the oncogenic program initiated by EWS-FLI-1, establishing a critical axis in the pathophysiology of ES [199].

MYC serves as a master regulator of key cellular processes, including proliferation, metabolism, and survival. Its transcriptional network governs a broad array of genes involved in cell cycle progression and ribosomal biogenesis. These processes are indispensable for the rapid and sustained growth of tumor cells. Notably, the interaction between EWS-FLI-1 and *MYC* extends beyond a mere regulatory relationship, as these two oncogenic drivers converge on overlapping sets of target genes. This convergence is particularly evident in genes involved in metabolic reprogramming and cell proliferation, creating a synergistic effect that further enhances the malignant potential of ES cells [200].

**Figure 28:**
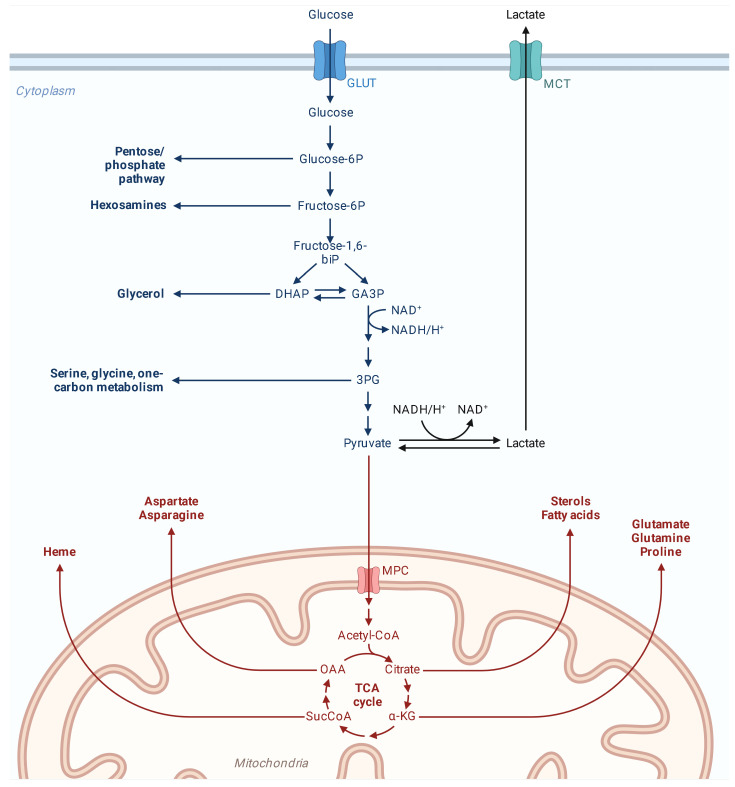
The Warburg effect, named after Otto Warburg, describes a phenomenon in which cancer cells display increased glucose uptake and preferentially utilize glycolysis even in the presence of oxygen. In the image, glucose enters the cell via GLUT transporters (blue), where it undergoes glycolysis, ultimately producing pyruvate. A significant portion of pyruvate is converted into lactate (also indicated in blue), which is exported out of the cell via MCT transporters. Although some pyruvate enters the mitochondria (illustrated in red) to fuel the TCA cycle, cancer cells favor glycolysis over oxidative phosphorylation. This metabolic shift not only provides rapid ATP generation but also generates essential metabolic intermediates required for biosynthetic pathways, such as nucleotide and lipid synthesis. The Warburg effect has implications for cancer diagnosis, treatment, and the understanding of tumor metabolism.

EWS-FLI-1 not only upregulates *MYC* but also reshapes the chromatin landscape, making it more permissive for MYC-mediated transcriptional activation. This collaboration facilitates metabolic adaptations, such as aerobic glycolysis (the Warburg effect), which supports the high energy demands and biosynthetic needs of aggressive tumor cells (Figure 28). By reprogramming both the transcriptome and the metabolome, the EWS-FLI-1/MYC axis creates a phenotype optimized for tumor survival, growth, and invasion [201].

The critical role of *MYC* in ES underscores its potential as a therapeutic target. However, MYC has long been considered “undruggable” due to its lack of a conventional binding pocket for small-molecule inhibitors. This has led to the exploration of indirect strategies to disrupt MYC function [202]. Additionally, efforts are underway to target kinases and other factors that stabilize MYC, aiming to destabilize its protein levels and diminish its oncogenic activity [203].

Moreover, targeting the functional synergy between EWS-FLI-1 and MYC could be a viable strategy. Disrupting EWS-FLI-1 activity with small-molecule inhibitors or RNA interference not only impairs its direct oncogenic functions but also reduces MYC expression, thus attenuating the downstream oncogenic cascade [55]. Additionally, metabolic therapies that exploit the vulnerabilities induced by MYC, such as inhibitors of glycolysis or glutaminase, have shown preclinical efficacy in MYC-driven cancers and are now being investigated in the context of ES. The interplay between EWS-FLI-1 and MYC represents a central driver of Ewing’s sarcoma pathogenesis. EWS-FLI-1 regulates MYC expression, while MYC amplifies the transcriptional and metabolic programs initiated by EWS-FLI-1, creating a self-reinforcing oncogenic loop. Targeting this interaction—either by disrupting EWS-FLI-1 activity or inhibiting MYC-dependent pathways—offers a promising avenue for therapeutic intervention in this aggressive cancer. Further research into these mechanisms will be critical for translating these insights into effective treatments for Ewing’s sarcoma patients.

## 9. MYC in Liposarcoma

**Figure 29:**
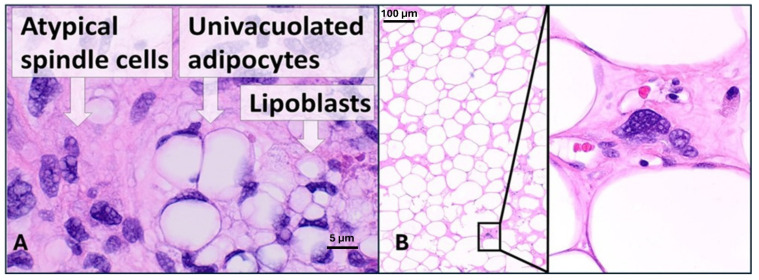
(**A**) Histopathology of liposarcoma, indicated by H&E stain, with the main features of spindle cells with enlarged, hyperchromatic nuclei, apparently unvacuolated adipocytes (may look normal), and lipoblasts (multivacuolated), but neither necessary nor sufficient for diagnosis [205]. (**B**) Histopathology of an atypical lipomatous tumor (also termed well-differentiated liposarcoma), lipoma-like subtype. At low magnification, the majority of the tumor has the look of benign mature adipocytes, but a high magnification of a fibrous band shows spindle cells with enlarged, hyperchromatic nuclei. H&E stain [205].

Liposarcoma (LS), a tumor of lipoblasts (Figure 29A), is a rare mesenchymal neoplasm that involves deep soft tissues, including the esophagus, retroperitoneum, and popliteal fossa [204].

The relative frequency of LS varies by tumor subtype and body location. For instance, dedifferentiated LS is predominantly found in retroperitoneal areas, while myxoid LS is more commonly observed in the lower extremities [206]. LS is extremely rare in the esophagus, where it typically presents as a slow-growing tumor, often affecting the upper part of the throat [207]. Most esophageal LS cases are well differentiated, localized, and carry a low risk of metastasis [208]; however, they have a relatively high local recurrence rate (10%), which can occur even up to 25 years after surgical resection [209].

Surgical excision is the mainstay of treatment [204]; wide and deep surgical excision, along with adjuvant radiation and/or chemotherapy, may be required for high-grade LSs. However, the response of LS to chemotherapy is not well established, making chemotherapy an experimental approach for this condition. Radiation therapy can be a useful complement to surgery, particularly for myxoid subtypes. In the case of esophageal LS, treatment options include minimally invasive endoscopic submucosal resection or, in more advanced cases, partial or total esophagectomy [210]. There are some case reports of using adjuvant radiation therapy to decrease the rate of recurrence. However, the role of adjuvant chemotherapy and radiation therapy remains controversial, and long-term follow-up is recommended due to the high recurrence rate associated with this condition [209].

The cause of LS remains unknown. Although the American Cancer Society has identified some potential risk factors for STS, many patients diagnosed with LS have no apparent risk factors. The specific genetic mutations associated with these malignancies are still under investigation. Known risk factors for LS include radiation exposure (particularly from radiation therapy for other cancers), certain familiar cancer syndromes, trauma or damage to the lymphatic system, and exposure to toxic chemicals. It is also important to emphasize that LSs do not develop from lipomas, which are entirely benign [211].

**Figure 30:**
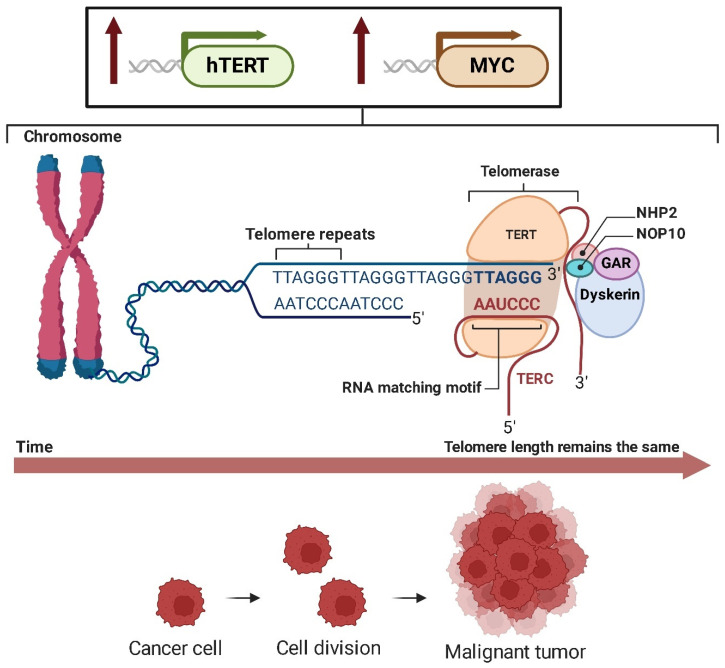
Increased expression of *hTERT* and *MYC* transcripts leads to the activation of telomerase, which is essential for maintaining telomere length and supporting tumor cell immortality. This enhanced telomerase activity is closely linked to tumor progression and is a marker of poor prognosis in LS.

As demonstrated by Schneider-Stock et al. [212], telomerase activity in LS is closely linked to the expression of the human telomerase reverse transcriptase (hTERT) and the *MYC* oncogene. Elevated levels of *hTERT* and *MYC* transcripts contribute to the activation of telomerase, which in turn plays a critical role in sustaining telomere length and promoting tumor cell immortality (Figure 30). This upregulation of telomerase activity is strongly associated with tumor progression and a poor prognosis in LS, underscoring its significance in the pathophysiology of the disease [213].

**Figure 31:**
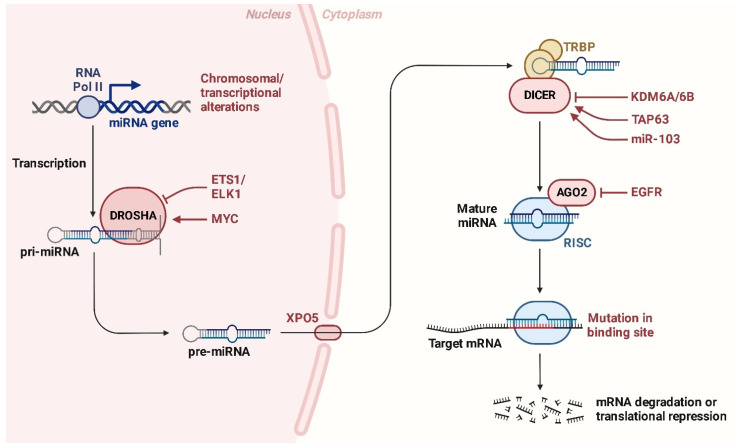
Biogenesis and function of miRNAs: miRNAs are first transcribed as precursor sequences (pre-miRNAs), which are processed into mature miRNAs. These mature miRNAs are then transported to the cytoplasm, where they bind to their target mRNAs, leading to gene silencing or degradation of the target transcript.

Another key aspect discussed by Zoroddu et al. [22], miRNAs, which play a crucial role in regulating tumorigenic processes, have emerged as potential therapeutic targets for LS treatment. In LS, the dysregulation of microRNAs (miRNAs) plays a critical role in tumorigenesis and progression. Tumor-suppressive miRNAs, including miR-143, miR-145, and miR-451, are often downregulated in LS. These miRNAs typically inhibit key oncogenic signaling pathways and promote cellular differentiation and apoptosis (Figure 31). Restoring the expression of these miRNAs has been shown to suppress tumor growth, induce differentiation, and enhance apoptosis, suggesting their potential therapeutic value.

On the other hand, oncogenic miRNAs such as miR-155 and miR-26a-2 are frequently overexpressed in LS. These miRNAs promote tumor progression by driving cellular proliferation, survival, and metastasis, thus contributing to the aggressive nature of the disease [214].

**Figure 32:**
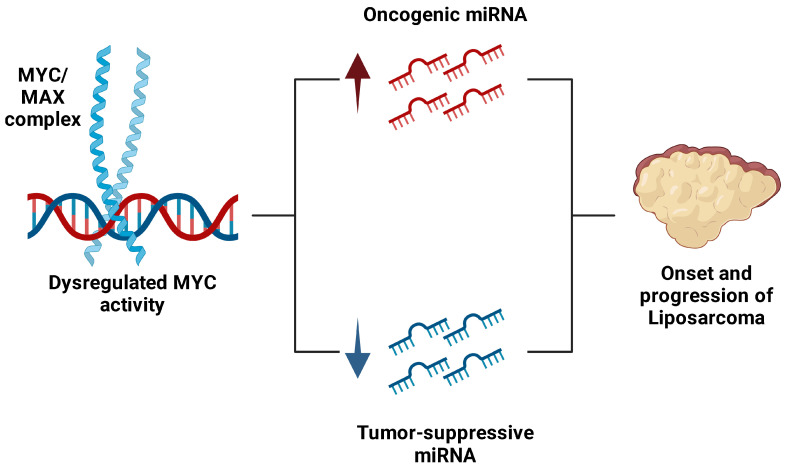
MYC plays a central role in regulating the expression of both tumor-suppressive and oncogenic miRNAs in LS. As a master transcriptional regulator, MYC influences the balance between miRNAs that inhibit tumor progression and those that promote it. Tumor-suppressive miRNAs such as miR-143, miR-145, and miR-451, which are often downregulated in LS, rely on MYC for their proper expression. On the other hand, MYC also drives the expression of oncogenic miRNAs like miR-155 and miR-26a-2, which are frequently overexpressed in LS. These miRNAs contribute to tumor progression, enhancing proliferation, survival, and metastasis. By regulating both types of miRNAs, MYC serves as a pivotal factor in the complex network of molecular processes underlying the onset and progression of LS.

The *MYC* oncogene plays a pivotal role in regulating both tumor-suppressive and oncogenic miRNAs, acting as a master transcriptional regulator of a diverse range of miRNAs involved in critical cellular processes. MYC directly influences the transcription of miRNAs that govern cell proliferation, differentiation, apoptosis, and metabolism. In LS and other cancers, dysregulated MYC activity disrupts the delicate balance between tumor-suppressive and oncogenic miRNAs (Figure 32). This leads to the suppression of miRNAs that would normally inhibit tumor growth, such as miR-145 and miR-143, while simultaneously enhancing the expression of oncogenic miRNAs like miR-155 and miR-26a-2, which drive proliferation and metastasis [215]. The aberrant expression of these miRNAs creates a tumor-promoting environment by activating oncogenic pathways and silencing genes that protect against tumorigenesis. This dysregulation not only facilitates unchecked tumor growth and invasion but also contributes to the aggressive and metastatic nature of LS. Understanding the role of MYC-mediated miRNA dysregulation offers a promising avenue for targeted therapeutic interventions aimed at restoring normal miRNA function and mitigating tumor progression [59].

Targeting MYC in LS may thus restore the balance of miRNAs and offer a novel therapeutic avenue, complementing strategies aimed at directly modulating miRNA levels. Given MYC’s involvement in tumor growth and metastasis, it stands out as a promising target for the development of targeted therapeutic strategies. Designing MYC-directed therapies could significantly improve treatment outcomes, offering a more effective approach to combating LS. As current treatment options remain limited and often ineffective, focusing on MYC as a therapeutic target could pave the way for innovative treatments that specifically address the underlying molecular mechanisms driving tumor progression, ultimately enhancing the survival and quality of life for patients.

## 10. MYC in Atypical Teratoid/Rhabdoid Tumor

**Figure 33:**
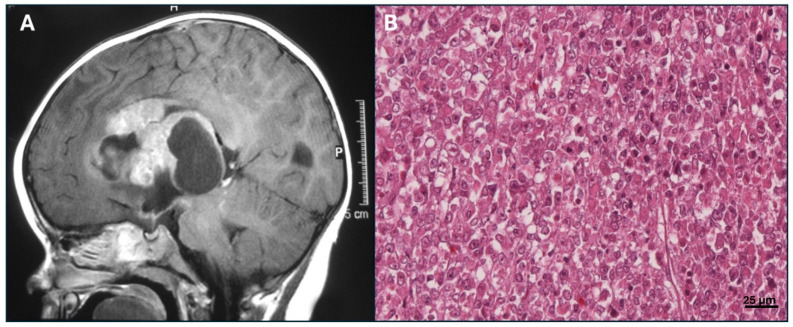
(**A**) This MRI image shows a sagittal view of the brain with a prominent, irregular mass located in the posterior fossa region. The lesion appears heterogeneous, with both solid and cystic components, indicating a complex internal structure. The solid portions of the tumor exhibit areas of contrast enhancement, which is suggestive of high vascularity and aggressive growth. The cystic components, on the other hand, represent areas of necrosis or fluid accumulation [143]. (**B**) The histological image displays a highly cellular tumor with a densely packed arrangement of cells. The cells exhibit marked pleomorphism, with noticeable variability in size and shape. The nuclei are large, irregular, and hyperchromatic, reflecting their aggressive nature. Evidence of mitotic activity is frequent, indicating rapid proliferation, and areas of necrosis may also be visible, suggesting high-grade malignancy. The stroma is sparse or nearly absent due to the tumor’s high cellularity [144].

The Atypical Teratoid/Rhabdoid tumor (AT/RT) is an aggressive central nervous system tumor that primarily affects infants, though it can also occur in older children and adults [216]. While AT/RT represents only 1–2% of all pediatric central nervous system (CNS) tumors, it is one of the more common malignant tumors in early childhood. Approximately 75% of AT/RT cases occur in children under the age of three. In this age group, AT/RT accounts for about 20% of embryonal CNS tumors and constitutes as much as 40–50% of all CNS malignancies diagnosed within the first year of life [217]. While AT/RT is rare in teenagers and adults, the median age at diagnosis falls between 16 and 30 months [218]. The tumor exhibits a slight male predominance, with a reported male-to-female ratio ranging from 1.1 to 2 [219]. AT/RT primarily occurs in the infratentorial region, though this location varies with age. In infants under one year old, tumors are predominantly found in the posterior fossa (Figure 33A). In toddlers, supratentorial tumors are more frequently observed, while spinal tumors are more common in children aged three years and older [220]. Additionally, metastatic spread is present at diagnosis in 20–40% of cases, although its impact on survival outcomes has been reported inconsistently [221].

The loss of SMARCB1, a critical subunit of the SWI/SNF chromatin-remodeling complex, represents the primary genetic event in the development of AT/RT [222]. This genetic alteration disrupts the function of the SWI/SNF complex, impairing chromatin remodeling and leading to a failure in proper cell differentiation. This disruption fosters an environment conducive to tumor progression.

**Figure 34:**
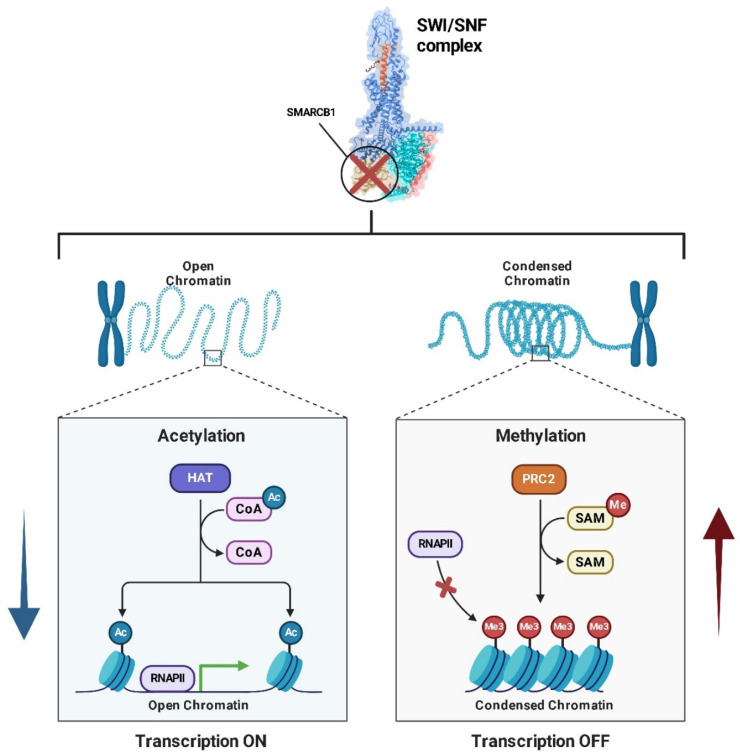
The SWI/SNF complex with SMARCB1 depletion can influence gene expression regulation through the deposition of different histone marks; it downregulates histone acetylation, performed by Histone Acetyl Transferases (HATs) by using Acetyl-CoA as a donor, which leads to chromatin opening that allows RNAPII to bind to a promoter to induce target gene expression. Conversely, it upregulates histone methylation, performed by Polycomb Repressive Complex 2 (PRC2) by using SAM as a donor, which leads to chromatin closing and represses transcription by preventing RNAPII binding.

One of the notable characteristics of AT/RTs is their striking genomic stability. Beyond the inactivation of SMARCB1, there are relatively few additional mutations observed in these tumors, emphasizing the central role of SMARCB1 loss in AT/RT pathogenesis [223]. The absence of SMARCB1 has profound downstream effects, including reduced H3K27 acetylation at critical enhancer regions. Additionally, it causes dysregulation of PRC2-mediated methylation, further promoting tumor growth and maintenance (Figure 34) [224]. Recent studies exploring the interplay between the SWI/SNF complex and PRC2 have identified novel therapeutic opportunities. One such promising approach involves targeting PRC2 with EZH2 inhibitors, which could counteract the tumor-promoting effects of SMARCB1 loss [225].

Genome-wide methylation profiling and RNA sequencing data in subsequent studies have revealed the existence of three molecular subtypes: Notch/sonic hedgehog (ATRT-*SHH*), tyrosinase enzyme (ATRT-*TYR*), and *MYC* oncogene (ATRT-*MYC*). These subgroups are distinguished by differences in gene expression profiles, tumor location, mechanisms of SMARCB1 inactivation, and their varying responsiveness to targeted therapies [226].

The ATRT-*SHH* subtype overexpresses components of the Notch and sonic hedgehog pathways. Gene set enrichment analyses revealed that ATRT-*SHH* is mainly a neuronally differentiated subtype [227]. The ATRT-*TYR* subtype overexpressed tyrosinase [228], which is essential for neural tube development [229]. The ATRT-*MYC* subtype exhibits excessive activation of the MYC pathway. The median age is significantly higher among patients with ATRT-*MYC* than among patients with the other subtypes of AT/RTs. Furthermore, most adult AT/RTs have been found to belong to the ATRT-*MYC* subgroup, and further clinical and molecular heterogeneity in ATRT-*MYC* may be revealed [230]. Alimova et al. [164], using both in vitro and in vivo studies, demonstrated that the *MYC* oncogene plays a pivotal role in regulating the malignant behavior of SMARCB1-deficient AT/RTs.

ATRT-*MYC* typically arises in older patients (median age 27 months) and is primarily located supratentorially, with all spinal AT/RT cases belonging to this subgroup [227].

Xenograft and mouse models resembling ATRT-*MYC* have been developed, offering promising platforms for drug testing and further research [231].

Given the rarity of AT/RT, no established “standard therapy” currently exists for its treatment, but recent studies highlight molecular similarities between ATRT-*MYC* and extracranial MRT, suggesting shared cellular origins and potential common therapeutic targets [232]. The development of MYC-focused therapies is particularly compelling given the oncogene’s central role in driving tumorigenesis across various cancers, including AT/RT. Targeting MYC as a therapeutic approach offers significant potential not only to enhance treatment outcomes for this rare tumor but also to drive broader progress in the field of oncology.

## 11. Conclusions and Future Perspective

**Figure 35:**
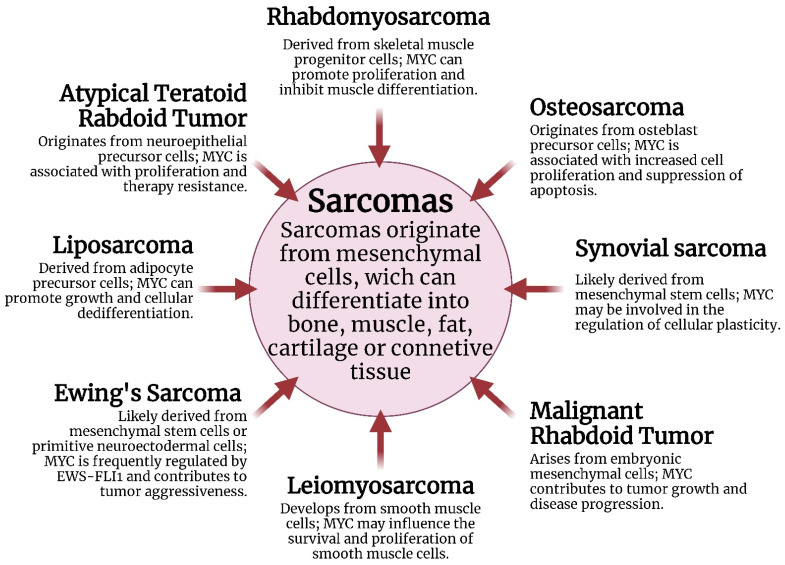
Overview of the main sarcoma subtypes and their cells of origin. Sarcomas arise from mesenchymal cells, which can differentiate into bone, muscle, fat, cartilage, or connective tissue. Major subtypes include Rhabdomyosarcoma (originating from skeletal muscle progenitors), osteosarcoma (from osteoblast precursors), liposarcoma (from adipocyte precursors), Leiomyosarcoma (from smooth muscle cells), synovial sarcoma (from mesenchymal stem cells with both mesenchymal and epithelial differentiation potential), Ewing’s sarcoma (likely derived from mesenchymal or primitive neuroectodermal cells), and Malignant or Atypical Teratoid/Rhabdoid tumors (from embryonic mesenchymal or neuroepithelial cells). This classification highlights the heterogeneity of sarcomas.

The *MYC* oncogene plays a pivotal role in the development and progression of various sarcoma subtypes, including RMS, OS, SyS, MRT, LMS, ES, LS, and AT/RT (Figure 35). Acting as a master transcriptional regulator, MYC drives processes such as tumor cell proliferation, survival, metabolic reprogramming, and metastasis by modulating a wide range of oncogenic pathways and miRNAs. Its dysregulation is a defining characteristic of these malignancies, underscoring its critical role in sarcoma pathophysiology.

Therapeutic strategies aimed at targeting MYC are a major focus of ongoing research. Current approaches involve the indirect inhibition of MYC activity by disrupting upstream signaling pathways, targeting transcriptional cofactors required for MYC function, and exploiting synthetic lethality to uncover vulnerabilities in MYC-driven tumors [233,234]. Furthermore, innovative techniques such as proteolysis-targeting chimeras (PROTACs) [235] and RNA-based therapeutics [236] have demonstrated significant anti-cancer effects across multiple tumor types.

One particularly exciting approach could be the use of the dominant-negative peptide OmoMYC^®^. This peptide inhibits MYC by preventing its dimerization and DNA binding [237] and has demonstrated significant anti-cancer effects across multiple tumor types [39].

Looking to the future, advancing MYC-targeted therapies will require optimizing delivery methods, improving tumor specificity, and combining MYC inhibitors with other treatments to enhance efficacy and reduce resistance. These developments could reshape the therapeutic options for MYC-driven sarcomas, offering renewed hope to patients facing these challenging malignancies.

## Figures and Tables

**Table 1:** Subtypes of RMS, each with its incidence, age of onset, origin, and genetic alteration.

Subtype	Incidence	Age of Onset	Origin	Genetic Alteration
Embryonal Rhabdomyosarcoma (ERMS)	The most common subtype (approximately 60–70% of cases) [22]	Predominantly occurs in children under 10 years of age	Arises from primitive mesenchymal tissues; frequently involves the head and neck region, or genitourinary tract.	Often associated with loss of heterozygosity (LOH) at 11p15.
Alveolar Rhabdomyosarcoma (ARMS)	Accounts for about 20–30% of cases [22]	Typically seen in adolescents and young adults (10–25 years)	Originates from skeletal muscle; it affects the extremities, trunk, and deep tissues.	Frequently characterized by *PAX3-FOXO1* or *PAX7-FOXO1* translocations.
Pleomorphic Rhabdomyosarcoma (PRMS)	Rare in children; more common in adults [23]	Primarily affects adults, especially those over 40 years of age	Develops from mature muscle tissue; often observed in the limbs or trunk.	Lacks specific recurrent genetic alterations; shows high genomic instability.
Spindle Cell/Sclerosing Rhabdomyosarcoma (SpRMS)	Rare (<5% of cases) [23]	Can occur at any age, but is more common in children and young adults	A variant of mesenchymal tissue; locations vary.	Associated with *MYOD1* mutations, especially in sclerosing subtypes.

**Table 2:** Most frequent gene mutations in osteosarcomas, with their incidence, phenotypic characteristic, and primary therapeutic approach.

Gene/Protein Involved	Type of Mutation	Incidence	Phenotypic Characteristic	Primary Therapeutic Approach
*RB* (*Retinoblastoma*)	Homozygous loss or altered gene product (pRb)	Common in osteosarcomas	Loss of cell cycle suppression	Surgery + standard chemotherapy (e.g., doxorubicin, cisplatin)
*p53*	Mutations in the gene	Frequent in carcinomas and bone/soft tissue sarcomas	Disruption of transcription regulation and cell cycle control	Surgery + chemotherapy; potential for MDM2 inhibitors in tumors with dysfunctional p53
*MDM2*	Amplification or overexpression	Observed in osteosarcomas	Inactivation of p53	Targeted therapies (e.g., MDM2 inhibitors, if available) + chemotherapy
*CDK4*	Amplification or overexpression	Observed in osteosarcomas	Phosphorylation and inactivation of pRb	CDK4/6 inhibitors combined with standard therapies
*MYC*	Overexpression	Demonstrated in osteosarcoma cell models	Enhanced tumor cell invasion	Experimental targeted therapies (if available) + surgery and chemotherapy

## Data Availability

All data relevant to the study are included in the article.
